# The primitive endoderm supports lineage plasticity to enable regulative development

**DOI:** 10.1016/j.cell.2024.05.051

**Published:** 2024-07-25

**Authors:** Madeleine Linneberg-Agerholm, Annika Charlotte Sell, Alba Redó-Riveiro, Marta Perera, Martin Proks, Teresa E. Knudsen, Antonio Barral, Miguel Manzanares, Joshua M. Brickman

**Affiliations:** 1 Novo Nordisk Foundation Center for Stem Cell Medicine (reNEW), University of Copenhagen, 2200 Copenhagen N, Denmark; 2 Centro de Biología Molecular Severo Ochoa (CBM), CSIC-UAM, 28049 Madrid, Spain

**Keywords:** Oct4/Pou5f1, plasticity, preimplantation development, totipotency, primitive endoderm, enhancer, JAK/STAT, hypoblast, transcription, pluripotency

## Abstract

Mammalian blastocyst formation involves the specification of the trophectoderm followed by the differentiation of the inner cell mass into embryonic epiblast and extra-embryonic primitive endoderm (PrE). During this time, the embryo maintains a window of plasticity and can redirect its cellular fate when challenged experimentally. In this context, we found that the PrE alone was sufficient to regenerate a complete blastocyst and continue post-implantation development. We identify an *in vitro* population similar to the early PrE *in vivo* that exhibits the same embryonic and extra-embryonic potency and can form complete stem cell-based embryo models, termed blastoids. Commitment in the PrE is suppressed by JAK/STAT signaling, collaborating with OCT4 and the sustained expression of a subset of pluripotency-related transcription factors that safeguard an enhancer landscape permissive for multi-lineage differentiation. Our observations support the notion that transcription factor persistence underlies plasticity in regulative development and highlight the importance of the PrE in perturbed development.

## Highlights

• The primitive endoderm can form an embryo in the absence of any other cell types
• Naive extra-embryonic endoderm (nEnd) stem cells recapitulate this capacity *in vitro*
• OCT4 expression in the primitive endoderm sustains plasticity
• The enhancer landscape in nEnd maintains plasticity for all preimplantation lineages

## Graphical abstract

**Figure:**
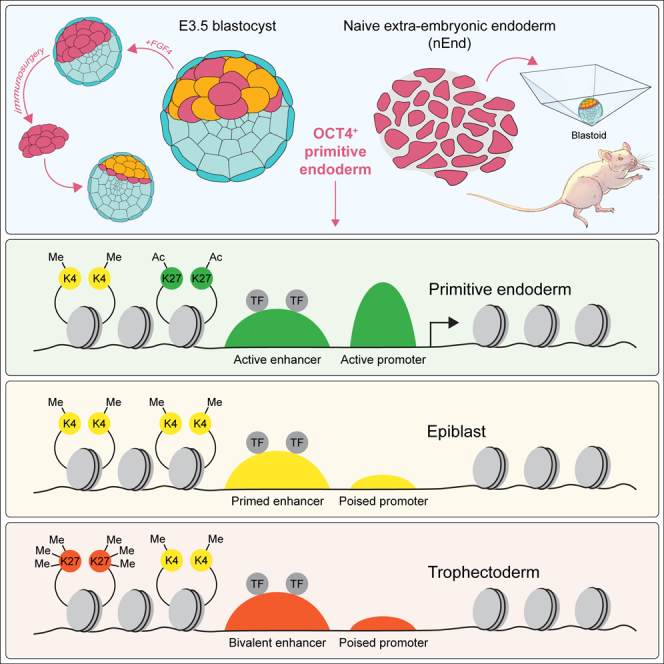

## Teaser

Although generally considered a simple support tissue, extra-embryonic primitive endoderm has the capacity to regenerate a complete blastocyst and continue post-implantation development.

## Introduction

Cells of the early embryo possess the remarkable ability to adapt and modulate their fate in response to perturbations. This feature is referred to as plasticity and enables cells to change their differentiation trajectory, a hallmark of regulative development. In mouse, the 2-cell stage blastomere is able to generate a complete organism from a single cell,^1^^,^^2^ after which the potential of each blastomere is then progressively restricted as development proceeds.^3^^,^^4^^,^^5^^,^^6^^,^^7^

Lineage specification begins after morula compaction with the acquisition of polarity in the outer cells that form the extra-embryonic trophectoderm (TE) surrounding the inner cell mass (ICM). Blastocyst formation follows alongside fibroblast growth factor/extracellular signal-regulated kinase (FGF/ERK)-mediated differentiation of ICM cells to epiblast (Epi), which gives rise to the embryo proper, or the extra-embryonic primitive endoderm (PrE, also known as hypoblast), which later forms the parietal endoderm (PE) and visceral endoderm (VE).^8^ When challenged experimentally, the PrE maintains a longer window of plasticity than the Epi,^9^^,^^10^^,^^11^ and during normal development, PrE-to-Epi cell fate switching is observed, but never vice versa.^12^^,^^13^ By the late blastocyst stage, this plasticity is lost and lineage commitment ensues.

Oct4/*Pou5f1* is a Pit-Oct-Unc (POU)-homeodomain transcription factor (TF) known for its role in supporting pluripotency *in vivo* and in embryonic stem cells (ESCs), as well as in TF-mediated reprogramming of somatic cells to induced pluripotent stem cells (iPSCs).^14^^,^^15^^,^^16^^,^^17^ Initially expressed throughout the embryo, OCT4 is then retained in both early Epi and PrE cells,^9^^,^^18^^,^^19^^,^^20^ where it is required cell autonomously during PrE specification.^18^ OCT4 is also required for PrE induction *in vitro*, where alternative partnering of OCT4 with SOX17 over SOX2 permits expression of endodermal genes.^21^ Following PrE specification, OCT4 is restricted to the Epi, coinciding with the loss of plasticity during blastocyst maturation.^9^

Naive ESCs are heterogeneous cell lines that recapitulate the ICM populations from which they are derived.^22^^,^^23^^,^^24^^,^^25^^,^^26^^,^^27^^,^^28^^,^^29^ These can be cultured in a variety of conditions,^30^ including serum-containing medium supplemented with the cytokine leukemia inhibitory factor (LIF) (S/L) and defined conditions with LIF and inhibitors of glycogen synthase kinase 3 (GSK3) and mitogen-activated protein kinase kinase (MEK) (2iLIF)^31^ supporting the so-called ground state of early Epi alongside a smaller population of ICM-like cells.^23^^,^^30^^,^^32^ Trophoblast stem cells (TSCs) recapitulating the late TE and early extra-embryonic ectoderm^33^ and extra-embryonic endoderm (XEN) cells that resemble the postimplantation-stage PE^34^^,^^35^ have similarly been reported. We previously described blastocyst-stage PrE stem cells derived from naive ESCs that can be expanded as naive extra-embryonic endoderm (nEnd) supported by LIF, Wnt, and transforming growth factor (TGF)-β signaling.^36^ Where ESC heterogeneities reflect differentiation competence, this is likely also a property of other stem cell models, including nEnd.

In this paper, we probe the molecular basis for cell plasticity in the preimplantation embryo in the context of regulative development and find that cells of the early PrE alone are sufficient to regenerate both Epi and TE. We recapitulate this plasticity *in vitro* using nEnd and demonstrate that an OCT4-expressing population in nEnd is competent to form Epi and TE upon targeted differentiation and during blastoid or chimera formation. Plasticity in the early extra-embryonic endoderm is supported by janus kinase/signal transducer and activation of transcription (JAK/STAT) signaling and occurs via OCT4-mediated safeguarding of transcriptionally quiescent enhancers.

## Results

### The E3.5 PrE maintains multi-lineage plasticity

**Figure 1:**
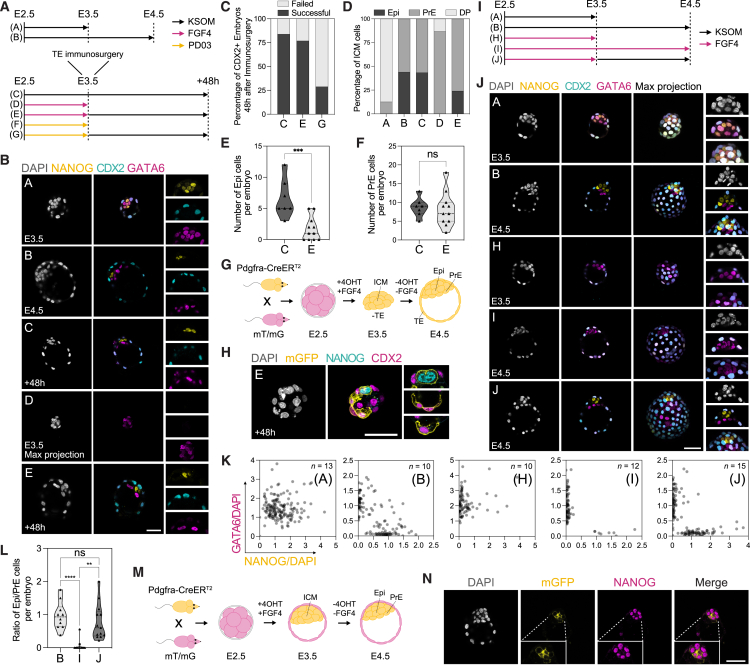
The E3.5 PrE reconstructs embryonic and extra-embryonic lineages following perturbation (A) Treatment regimen that 8-cell embryos were subjected to in (B)–(F). (B) Immunostaining of embryos for indicated markers after treatment. (C) Embryo reconstruction following immunosurgery based on presence of CDX2-expressing reconstructed TE. (D) ICM lineage allocation in control and treated embryos. (E and F) Allocation of E Epi and F PrE cells per ICM in control and treated embryos. (G) Schematic of treatment regimen that 8-cell PdgfraCre-ER^T2^:Rosa26^mT/mG^ embryos were subjected to in (H). (H) Immunostaining of PdgfraCre-ER^T2^:Rosa26^mT/mG^ embryos at E4.5 + 48 h for indicated markers. (I) Treatment regimen that 8-cell embryos were subjected to in (J)–(L). (J) Immunostaining of E4.5 embryos for indicated markers after treatment. (K) Single-cell quantification of embryos for NANOG and GATA6 immunostaining normalized to DAPI. *n* values indicate total number of embryos quantified. (L) Ratio of Epi to PrE cells per embryo. (M) Treatment regimen subjected to 8-cell PdgfraCre-ER^T2^:Rosa26^mT/mG^ embryos in (N). (N) Immunostaining of E4.5 PdgfraCre-ER^T2^:Rosa26^mT/mG^ embryos for indicated markers. *n* values for treatment regimens outlined in (A), (G), (I), and (M) in Table S1. *p* values determined by unpaired t test. Scale bars, 50 μm. See also Figure S1.

**Figure S1:**
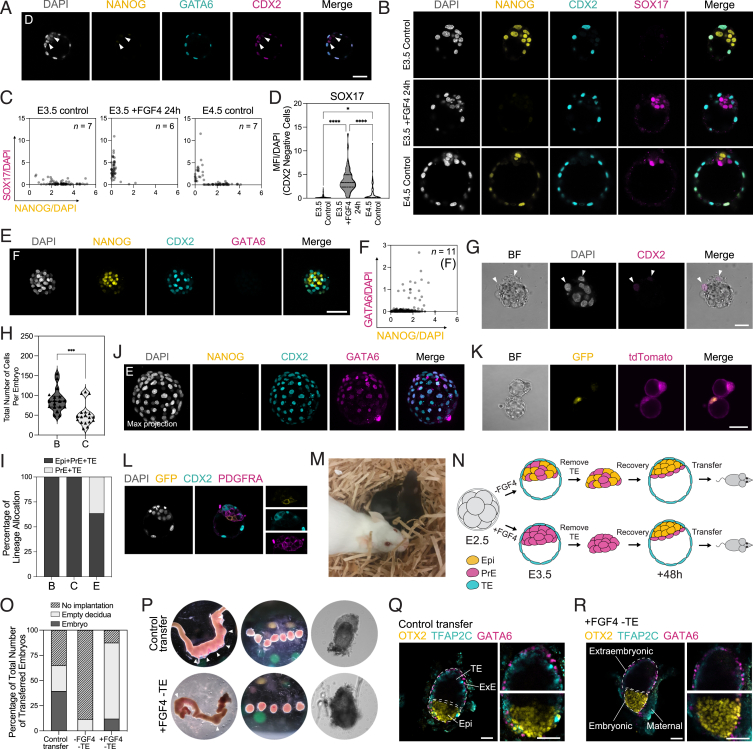
Quantification of control and treatment conditions, related to Figure 1 (A) Immunostaining of an E3.5 embryo treated with FGF4 for 24 h for indicated markers. White arrowheads indicate cells expressing GATA6 and low levels of NANOG. *n* values in Table S1. (B) Immunostaining of an E3.5 embryo treated with FGF4 for 24 h alongside a control E3.5 and E4.5 embryo for indicated markers. (C) Single-cell quantification of embryos in (B) for SOX17 and NANOG immunostaining normalized to DAPI. *n* values indicate total number of embryos quantified. (D) Quantification of total expression of SOX17 of embryos in (B). *n* values as in (C). (E) Immunostaining of an E3.5 embryo treated with PD03 for 24 h for indicated markers. (F) Single-cell quantification of embryos in (E) for NANOG and GATA6 immunostaining normalized to DAPI. *n* values indicate total number of embryos quantified. (G) Immunostaining of an embryo 48 h following immunosurgery for CDX2 and DAPI. White arrowheads indicate morphologically stressed cells expressing CDX2. (H) Quantification of total number of cells per embryo based on DAPI in embryos subjected to treatment outlined in Figure 1A, where (B) *n* = 18 embryos; (C) *n* = 18 embryos. (I) Lineage allocation of E4.5 embryos following immunosurgery in control and FGF4-treated embryos. (J) Immunostaining of an embryo subjected to treatment (E) outlined in Figure 1A for indicated markers. (K) Bright-field and immunofluorescence imaging of E3.5 PdgfraCre-ER^T2^:Rosa26^mT/mG^ embryos following FGF4 and 4OHT treatment for 24 h, imaged by widefield microscopy. (L) Immunostaining of embryos in (K) for indicated markers (*n* = 4 embryos). (M) Photograph of F_1_ pups derived from E3.5 PrE detailed in Figure 1I, where FGF4-treated embryos were transferred to pseudopregnant females at E3.5. Male offspring were later mated, demonstrating germline transmission. (N) Workflow to establish viability of embryos at E6.5 following trophectoderm reconstruction in FGF4-treated embryos compared with control. (O) Embryo survival and developmental progression across indicated treatments. (P) Bright-field images of uterine horns (left), deciduae (middle), and embryos (right) for control transfers and FGF4-treated embryos. White arrowheads indicate position of deciduae within uterine horn. (Q and R) Immunostaining of (Q) control and (R) treated E6.5 embryos for indicated markers, where dashed white lines indicate embryonic and extra-embryonic regions. *p* values determined by unpaired t test, and error bars represent ± SD. Scale bars, 50 μm.

The regulative properties of the mammalian preimplantation embryo have been shown by numerous grafting experiments, including regeneration of the TE from an isolated ICM at the mid-blastocyst stage.^4^ To determine the lineage responsible for this regeneration, we converted ICMs into either PrE or Epi by treatment of 8-cell stage embryos with FGF4 or the MEK inhibitor PD0329501 (PD03), respectively, for 24 h,^11^^,^^37^ after which we removed the TE by immunosurgery (condition D and F)^38^ (Figure 1A). To confirm that treated ICMs were homogenously PrE or Epi, we quantified cells expressing the PrE-marker GATA6 and the Epi-marker NANOG. In FGF4-treated embryos (condition D), we found that 76.9% of embryos were entirely single positive for GATA6, where the remaining 23.1% of embryos contained few cells expressing high levels of GATA6 with low levels of NANOG (Figures 1B and S1A; Table S1), consistent with previous studies.^39^ There were no embryos containing NANOG single positive cells. PrE marker expression downstream of GATA6 showed that all cells within the ICM of FGF4-treated embryos expressed high levels of SOX17 (Figures S1B–S1D). As SOX17 is a unique PrE marker not normally expressed at these levels until later stages,^40^^,^^41^ this suggests that FGF4 is both converting the ICM to PrE while also stimulating PrE differentiation. In PD03-treated embryos (condition F), 81.2% were single positive for NANOG (Figures S1E and S1F; Table S1). Quantification of CDX2 expression in isolated ICMs across all conditions demonstrated no CDX2-positive cells immediately following immunosurgery in most embryos, and the few that did (14%), contained ≤2 CDX2-positive cells with significant damage to their membrane integrity, indicating that these are likely not functional (Figure S1G). Following 48 h of recovery, the TE was reconstructed *de novo* in 79.2% of control embryos (condition C) and 63.6% of embryos treated with FGF4 (condition E), but only 29% of embryos treated with PD03 (condition G) based on quantification of immunostaining for the TE marker CDX2 (Figures 1B and 1C; Table S1). Consistent with previous embryo-scaling studies,^2^^,^^39^^,^^42^^,^^43^ embryos subjected to immunosurgery produced smaller blastocysts with significantly lower total number of cells (Figure S1H).

Although all control embryos formed blastocysts containing NANOG (Epi)-, GATA6 (PrE)-, and CDX2 (TE)-expressing cells at normal proportions, this occurred in only 63.6% of FGF4-treated embryos (condition E), where 36.4% of these instead formed blastocysts containing only CDX2- and GATA6-positive cells (Figures 1B, 1D, S1I, and S1J). When quantifying the allocation of ICM cells in the embryos that did successfully specify an Epi, FGF4-treated embryos also contained significantly less Epi cells per ICM compared with control embryos (Figure 1E), while establishing normal PrE cell numbers (Figure 1F). These results suggest that, in a context where a PrE cell is faced with the decision become either Epi or TE, it prioritizes TE.

To further ensure that the PrE is giving rise to Epi and TE, we performed lineage tracing with a Pdgfra-CreER^T2^ heterozygous mouse line^44^ crossed with homozygous Rosa26^mT/mG^ mice^45^ to drive 4-hydroxytamoxifen (4OHT)-induced membrane GFP (mGFP) expression in cells upregulating the PrE-specific marker PDGFRA,^12^^,^^46^ with constitutive expression of membrane tdTomato where mGFP is absent. We collected embryos at E2.5 and treated these with FGF4 and 4OHT for 24 h (Figure 1G), where at E3.5 there was mGFP expression specifically within the ICM (Figure S1K). Consistent with previous reports,^47^ Cre-mediated recombination heterogeneously labeled only a subset of PDGFRA-expressing cells (Figure S1L). The TE was then removed by immunosurgery followed by recovery for 48 h (Figure 1G). In reconstructed blastocysts, we found CDX2 and NANOG co-expressed with mGFP (Figure 1H; Table S1), demonstrating that a PrE cell previously expressing PDGFRA changed identity to Epi or TE.

To determine whether the reduced capacity for Epi regeneration (Figure 1E) is based on the competence of the PrE or the limited capacity of these cells to regenerate both Epi and TE lineages simultaneously, we again treated 8-cell embryos with FGF4 for 24 h and asked whether ICMs containing only PrE could generate Epi in the context of an intact TE when released from FGF4 stimulation (Figure 1I). The majority of embryos (89%) contained NANOG single positive cells after treatment, with total levels of GATA6 and NANOG expression similar to the control embryos (condition J) (Figures 1J and 1K; Table S1) and the ratio of Epi/PrE cells per embryo in FGF4-treated embryos similar to the controls (Figure 1L). When crossing Pdgfra-CreER^T2^ and Rosa26^mT/mG^ mice and treating 8-cell embryos with FGF4 and 4OHT for 24 h (Figure 1M), after 24 h of recovery these E4.5 embryos also contained NANOG and mGFP co-expressing cells (Figure 1N). Treatment of wild-type 8-cell embryos with FGF4 for 24 h followed by transfer to pseudopregnant mice produced live births and germline transmission (Figure S1M), confirming that the E3.5 PrE maintains competence for both Epi differentiation and fetal development where offspring maintain germline competence.

Having established that an immunosurgery-isolated ICM composed entirely of GATA6-positive PrE is able to regenerate both TE and Epi, we reasoned that the PrE prioritizes making a functional TE first to ensure implantation, after which specification of Epi will follow at a delayed rate. To test the functionality of *de novo* TE, we transferred successfully reconstructed FGF4-treated and control embryos to pseudopregnant mice (Figure S1N). Although the reconstructed embryos without FGF4 treatment produced only a single empty decidua, the treated embryos underwent extensive decidualization and could support normal development based on expression of OTX2 (Epi), GATA6 (VE/PE), and TFAP2C (extra-embryonic ectoderm) at E6.5 (Figures S1O–S1R).

### Naive extra-embryonic endoderm creates a pluripotent niche upon self-organization

**Figure S2:**
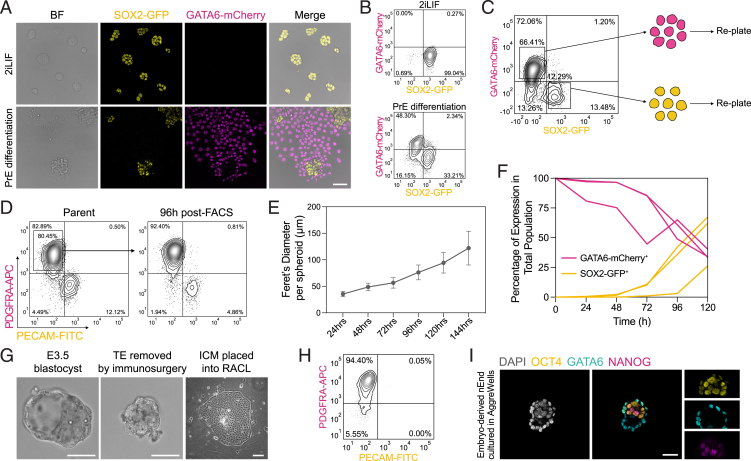
PrE and nEnd differentiation competence of SGGC reporter cells and derivation from E3.5 ICMs, related to Figure 2 (A) Bright-field and immunofluorescence imaging of SGGC reporter cells in 2iLIF and day 7 of PrE differentiation. (B) Flow cytometry contour plots of (A). Bottom left quadrant: gating based on a negative control. (C) Flow cytometry contour plots of SGGC nEnd showing gating strategy for isolating GATA6-mCherry^+^ and SOX2-GFP^+^ populations for re-plating. (D) Flow cytometry contour plots of E14JU nEnd stained for PDGFRA-APC and PECAM-FITC followed by FACS for PDGFRA-APC^+^ cells and analyzed by flow cytometry after 96 h. Bottom left quadrant: gating based on a negative control. (E) Quantification of 3D nEnd growth in size 24–144 h following FACS and seeding in AggreWells (*n* = 200 spheroids per time point). (F) Quantification of total expression by flow cytometry of SGGC 3D nEnd 0–120 h following FACS for GATA6-mCherry^+^/SOX2-GFP^−^ cells. (G) Bright-field images of nEnd derivation from E3.5 blastocysts. (H) Flow cytometry contour plots of embryo-derived nEnd stained for PDGFRA-APC and PECAM-FITC. Bottom left quadrant: gating based on a negative control. (I) Immunostaining of 3D nEnd from embryo-derived nEnd for indicated markers. Errors bars represent ± SD. Scale bars: 50 μm in (A) and (I); 100 μm in (G).

**Figure 2:**
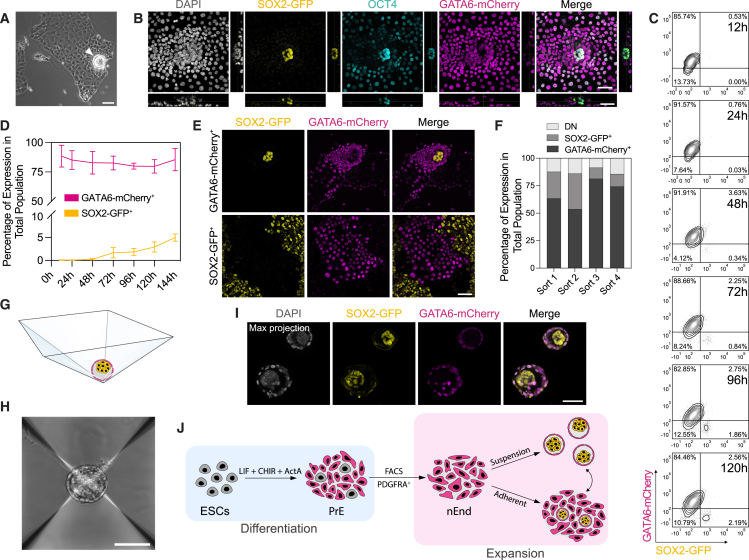
GATA6-expressing nEnd spontaneously undergoes de-differentiation to SOX2-expressing Epi-like cells (A) Bright-field image of nEnd with aggregate emerging from monolayer (white arrowhead). (B) Immunostaining of SGGC nEnd for OCT4 and DAPI with orthogonal projection. (C) Flow cytometry contour plots of SGGC nEnd 12–120 h following FACS for GATA6-mCherry^+^/SOX2-GFP^−^ cells (*n* = 4 biological replicates). Bottom left quadrant: gating based on a negative control. (D) Quantification of total expression by flow cytometry of SGGC nEnd 12–144 h following FACS for GATA6-mCherry^+^/SOX2-GFP^−^ cells (*n* = 4 biological replicates). (E) Immunofluorescence imaging of SGGC nEnd 7 days following isolation by FACS for GATA6-mCherry^+^ or SOX2-GFP^+^ cells. (F) Quantification of total expression of SGGC nEnd subpopulations across 4 consecutive rounds of FACS for GATA6-mCherry^+^ nEnd. Time points collected 5 days after seeding. (G) Illustration of 3D nEnd cultured in AggreWells with endodermal outside cells (magenta) and Epi-like inside cells (yellow). (H) Bright-field image of 3D nEnd cultured in AggreWells. (I) Immunofluorescence imaging of SGGC 3D nEnd cultured in AggreWells. (J) Schematic of nEnd culture system. ESCs are differentiated toward PrE and following FACS for PDGFRA-APC expression, these are expanded as nEnd in adherent culture or 3D nEnd in suspension culture. Scale bars: 100 μm in (A) and (H); 50 μm in (B), (E), and (I). See also Figure S2.

To recapitulate the dynamic nature of the early PrE *in vitro*, we exploited a double reporter ESC line for SOX2-GFP for Epi and GATA6-mCherry for endoderm (SGGC).^48^ Following PrE differentiation (Figures S2A and S2B) and fluorescence-activated cell sorting (FACS) for GATA6-mCherry single positive cells, these were expanded as nEnd in RPMI-based defined medium containing Activin A, CHIR99021, and LIF (RACL).^36^^,^^49^^,^^50^ Throughout nEnd culture, we observed sporadic spheroid aggregates arising from the underlying endodermal monolayer (Figure 2A), composed of a GATA6-mCherry-positive outer layer and a SOX2-GFP-positive inner core (Figure 2B). To confirm that these aggregates were arising entirely from GATA6-mCherry-expressing nEnd, we isolated GATA6-mCherry single positive cells by FACS (Figure S2C) and found that a SOX2-GFP-positive population arose *de novo* within 48–72 h after seeding (Figures 2C and 2D). Similarly, in PDGFRA-positive nEnd generated from E14JU ESCs, a pluripotent population arose with the same kinetics based on expression of the ESC marker PECAM^51^ (Figure S2D). When re-plating nEnd-derived SOX2-GFP cells, these differentiated to GATA6-mCherry-expressing endoderm (Figure 2E). We confirmed that these were not a product of contaminating ESCs left over from differentiation by performing consecutive rounds of FACS to isolate pure GATA6-mCherry nEnd (Figure 2F).

To generate reproducible structures that supported this cell state transition, we used suspension culture in AggreWells (Figure 2G), referred to as three-dimensional (3D) nEnd, producing spheroids with GATA6-mCherry-positive outside cells and SOX2-GFP-positive inside cells abutted to a cavity (Figures 2H, 2I, S2E, and S2F). To determine whether a similar nEnd culture could be captured directly from mouse embryos, we removed the TE from E3.5 blastocysts by immunosurgery and cultured ICMs in RACL (Figure S2G). During initial passages, these cells were homogenously PDGFRA-APC-positive (Figure S2H). However, upon culture in AggreWells, embryo-derived nEnd formed 3D nEnd spheroids with a GATA6-positive outer layer and NANOG/OCT4-positive de-differentiated core (Figure S2I). Taken together, nEnd appears dynamically heterogeneous, containing a subpopulation capable of self-renewal and self-organization into delaminated Epi- and PrE-like cell types (Figure 2J).

### nEnd contains an Oct4-expressing population with enhanced developmental potential

**Figure S3:**
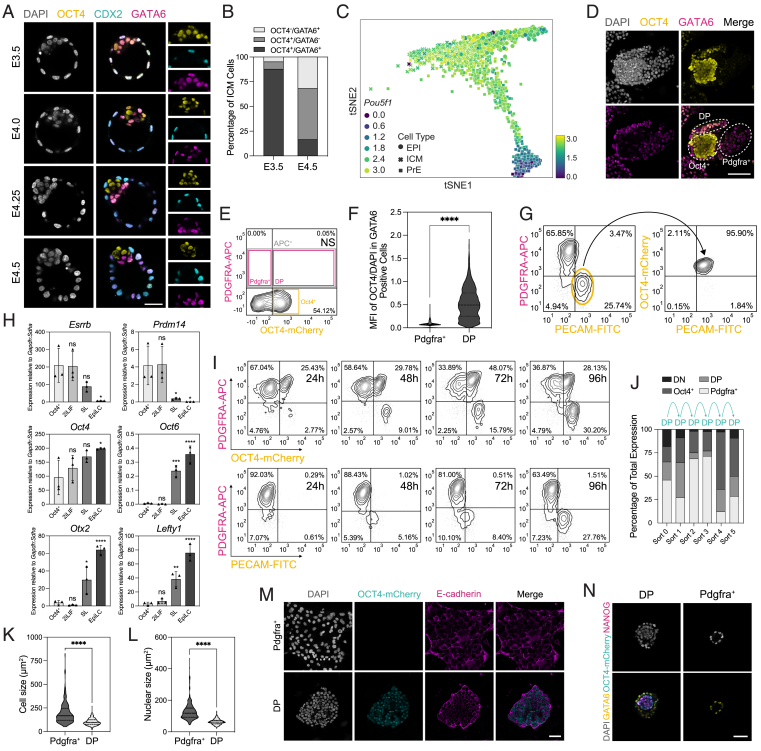
OCT4 expression separates uncommitted from committed extra-embryonic endoderm cell types, related to Figure 3 (A) Immunostaining of E3.5–E4.5 embryos for indicated markers, where E3.5: *n* = 20 embryos; E4.0: *n* = 5 embryos; E4.25: *n* = 5 embryos; E4.5: *n* = 17 embryos. (B) Quantification of E3.5 and E4.5 embryos for OCT4 and GATA6 expression, where E3.5: *n* = 20 embryos and 107 cells; E4.5: *n* = 17 embryos and 179 cells. (C) t-distributed stochastic neighbor (tSNE) embedding of scRNA-seq of the mouse preimplantation embryo at E3.5–4.5,^53^ where color scale represents expression of *Pou5f1* transcripts and shape indicates cell type. (D) Immunostaining of E14JU nEnd for indicated markers. (E) Flow cytometry contour plot of unstained OCT4-mCherry nEnd. Bottom left quadrant: gating based on a negative control. (F) Median fluorescence intensity (MFI) of OCT4 expression by immunostaining normalized to DAPI (*n* = 1,495 cells [left] and 1,467 cells [right]). (G) Flow cytometry contour plot of OCT4-mCherry nEnd stained for PECAM-FITC and PDGFRA-APC, where gating of PECAM-FITC^+^ cells (left) are visualized for OCT4-mCherry and PECAM-FITC co-expression (right). (H) RT-qPCR of Oct4^+^ isolated by FACS, 2iLIF, serum/LIF (SL), and EpiLC ESCs for indicated markers. (I) Flow cytometry contour plots for monitoring OCT4-mCherry, PECAM-FITC, and PDGFRA-APC expression from 24 to 96 h following FACS for PDGFRA-APC^+^ cells. Bottom left quadrant: gating based on a negative control. (J) Quantification of total percentage of expression of OCT4-mCherry-positive (Oct4^+^), double negative (DN), PDGFRA-APC single-positive (Pdgfra^+^), and double positive (DP) nEnd, based on flow cytometry following multiple consecutive rounds of FACS to isolate and expand the DP population. (K and L) Quantification of (K) cell size of Pdgfra^+^ (*n* = 333 cells) and DP (*n* = 511 cells) nEnd based on E-cadherin immunostaining and (L) nuclear size of Pdgfra^+^ (*n* = 426 cells) and DP (*n* = 400 cells) nEnd based on DAPI localization. Nuclear/cytoplasmic ratio: DP = 0.63; Pdgfra^+^ = 0.67. (M) Immunostaining of OCT4-mCherry nEnd for indicated markers. (N) Immunostaining of 3D nEnd cultured in AggreWells for indicated markers. *p* values determined by unpaired t test, and error bars represent ± SD. Scale bars, 50 μm.

**Figure 3:**
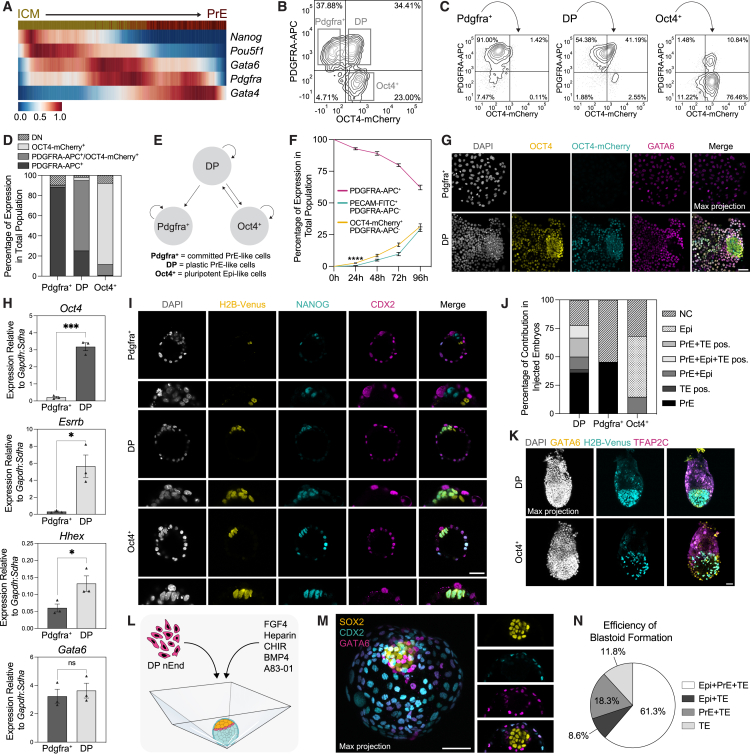
OCT4/PDGFRA co-expressing nEnd represents uncommitted PrE capable of multi-lineage differentiation (A) Heatmap of scaled expression of indicated genes from scRNA-seq of the mouse preimplantation embryo^53^ across scVelo-defined latent time. Top bar: progression from E3.5 ICM (dark yellow) to E4.5 PrE (dark red). (B) Flow cytometry contour plot of OCT4-mCherry nEnd stained for PDGFRA-APC. Boxes highlight Pdgfra^+^, DP, and Oct4^+^ populations. Bottom left quadrant: gating based on a negative control. (C) Flow cytometry contour plots of Pdgfra^+^, DP, and Oct4^+^ nEnd 5 days following FACS for PDGFRA-APC^+^ cells. Bottom left quadrant: gating based on a negative control in Figure S3E. (D) Quantification of (C) for subpopulation composition in total population for Pdgfra^+^, DP, and Oct4^+^ nEnd (DN, double negative). (E) Schematic of dynamic equilibrium established in nEnd culture. (F) Quantification of total expression by flow cytometry of OCT4-Cherry nEnd 0–96 h following FACS for PDGFRA-APC^+^ cells in Figure S3I. (G) Immunostaining of OCT4-mCherry nEnd for indicated markers 5 days following FACS for PDGFRA-APC^+^ cells. (H) RT-qPCR of Pdgfra^+^ and DP nEnd isolated by FACS for indicated markers. (I) Immunostaining of E4.5 embryos injected with either OCT4-mCherry-H2B-Venus Pdgfra^+^ (top), DP (middle), or Oct4^+^ (bottom) nEnd at the 8-cell stage for indicated markers. (J) Allocation of lineage contribution of OCT4-mCherry-H2B-Venus Pdgfra^+^ (*n* = 44 embryos), DP (*n* = 36 embryos), and Oct4^+^ (*n* = 25 embryos) nEnd in chimera embryos (NC, no contribution; TE pos., TE position). (K) Immunostaining of E6.5 embryos following clonal injection of OCT4-mCherry-H2B-Venus DP (top, *n* = 4 embryos) or Oct4^+^ (bottom, *n* = 3 embryos) cells at the 8-cell stage for indicated markers. (L) Schematic of blastoid generation^57^ from DP nEnd. (M) Immunostaining of a DP nEnd-derived blastoid for indicated markers. (N) Quantification of efficiency of blastoid formation defined as the presence of TE, PrE, and Epi-like cell types (*n* = 304 individual blastoids/trophospheres). *p* values determined by unpaired t test, and error bars represent ± standard deviation (SD). Scale bars, 50 μm. See also Figures S3–S5.

Although OCT4 is generally considered a pluripotency factor, it is widely expressed during PrE specification^9^^,^^18^^,^^19^^,^^20^^,^^52^ (Figures S3A and S3B). To further map *Oct4* expression during preimplantation development, we used available embryo single-cell RNA-sequencing (scRNA-seq) data^53^ (Figure S3C), referred to as Nowotschin et al.,^53^ and assessed differentiation trajectory with RNA velocity^54^^,^^55^^,^^56^ (Figure 3A). Although *Nanog* is rapidly downregulated before the first PrE progenitors, *Oct4* follows *Gata6* and *Pdgfra* expression throughout early PrE specification. *Oct4* is then gradually downregulated concurrently with upregulation of the late PrE marker *Gata4*.

Consistent with nEnd trapping a preimplantation PrE-like state, OCT4 was heterogeneously expressed throughout GATA6-positive nEnd culture organized into distinct patches (Figure S3D). To explore nEnd dynamics, we employed an OCT4-mCherry protein-based reporter cell line^58^ and by flow cytometry observed three populations based on co-expression with PDGFRA-APC: PDGFRA-APC single positive (Pdgfra^+^), PDGFRA-APC/OCT4-mCherry double positive (DP), and OCT4-mCherry single positive (Oct4^+^) (Figures 3B, S3E, and S3F). Here, Pdgfra^+^ and DP comprise the endodermal portion of nEnd culture, while Oct4^+^ represent the de-differentiated pluripotent cells based on co-expression with PECAM-fluorescein isothiocyanate (FITC) (Figure S3G). When compared with different ESC conditions along the spectrum of naive-to-primed pluripotency,^59^ these Oct4^+^ cells appear transcriptionally similar to naive 2iLIF and S/L ESCs (Figure S3H).

To determine the dynamic properties of nEnd, we isolated individual populations by FACS and re-plated them in adherent nEnd culture. After 120 h, DP nEnd were able to give rise to themselves as well as the other two populations, whereas Pdgfra^+^ cells remained PDGFRA-APC single positive, with a small population becoming double negative (DN) (Figures 3C–3E). Oct4^+^ nEnd were similarly able to self-renew, in addition to differentiation toward DP cells. When monitoring re-plated DP nEnd at 24 h intervals, single OCT4-mCherry expression in the de-differentiated population precedes upregulation of PECAM (Figures 3F and S3I). Following multiple consecutive rounds of FACS, DP nEnd were consistently able to re-establish all populations of nEnd culture (Figure S3J).

DP and Pdgfra^+^ nEnd are also morphologically distinct. Although they share similar nuclear/cytoplasmic size ratios, DP cells form compact colonies with overall cell and nuclei size that are significantly smaller than Pdgfra^+^ cells, which instead form large epithelial sheets (Figures S3K–S3M).

In adherent nEnd culture, DP cells form OCT4 single positive dome-shaped colonies surrounded by endodermal monolayers, whereas Pdgfra^+^ cells form only a GATA6 single positive monolayer (Figure 3G). Similarly, in 3D nEnd culture, only DP cells form Epi-PrE spheroids, with Pdgfra^+^ cells forming GATA6 single positive hollow spheres (Figure S3N). RT-qPCR revealed that DP cells express significantly higher levels of *Oct4*, *Esrrb*, and *Hhex* compared with Pdgfra^+^ cells, pointing toward an earlier uncommitted PrE identity,^25^^,^^56^^,^^60^ while expressing similar levels of *Gata6* (Figure 3H).

**Figure S4:**
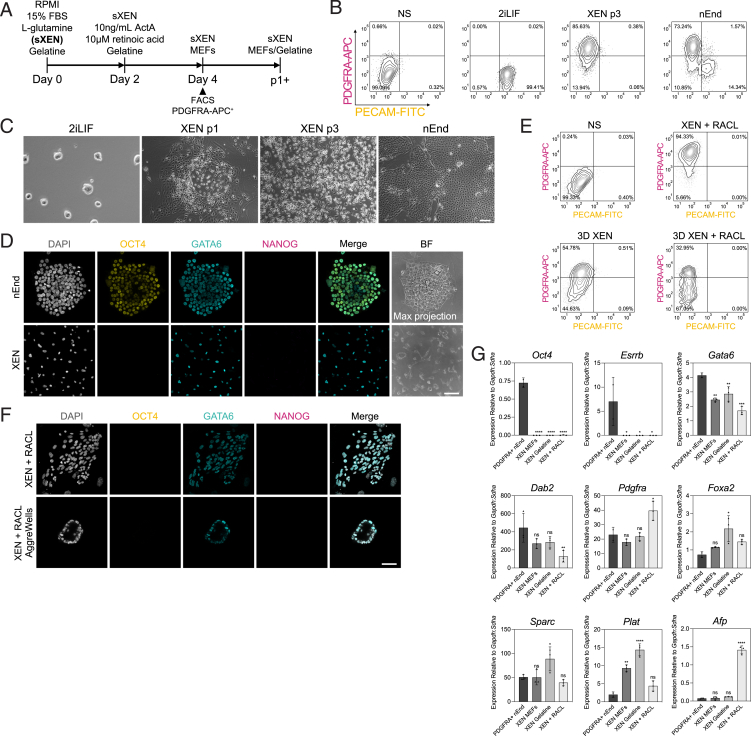
XEN cells represent later-stage extra-embryonic endoderm lacking OCT4 expression that cannot be rescued upon culture in nEnd medium, related to Figure 3 (A) Schematic of E14JU ESCs in 2iLIF toward XEN cells,^34^^,^^61^ followed by FACS for PDGFRA-APC^+^ cells to purify endoderm population. (B) Flow cytometry contour plots of XEN cell derivation compared with 2iLIF ESCs and nEnd stained for PECAM-FITC and PDGFRA-APC. Bottom left quadrant: gating based on a no stain (NS) negative control. (C) Bright-field images of XEN derivation compared with 2iLIF ESCs and nEnd. (D) Immunostaining and bright-field images of nEnd and XEN cells for indicated markers. (E) Flow cytometry contour plots of XEN cells cultured in indicated conditions stained for PECAM-FITC and PDGFRA-APC expression. Bottom left quadrant: gating based on a negative control. (F) Immunostaining of cXEN cells for indicated markers. (G) RT-qPCR of XEN cells compared with nEnd isolated by FACS for PDGFRA-APC expression for indicated markers. *p* values determined by unpaired t test, and error bars represent ± SD. Scale bars: 100 μm in (C); 50 μm in (D) and (F).

To benchmark nEnd culture within the developmental progression of extra-embryonic endoderm, we generated XEN cells from 2iLIF ESCs^34^^,^^61^ (Figures S4A–S4C). XEN cells appeared refractile by morphology (Figure S4C) and expressed GATA6, but not OCT4,^35^ (Figure S4D) and did not undergo de-differentiation based on PECAM-FITC expression (Figure S4E). XEN cells transferred to RACL in both adherent and suspension culture also failed to upregulate unique nEnd markers (Figures S4E–S4G), including *Oct4* and *Esrrb*, and instead expressed high levels of the VE marker *Afp*, suggesting XEN cells represent a more committed extra-embryonic endodermal cell type (Figure S4G).

### nEnd exhibits enhanced lineage competence

**Figure S5:**
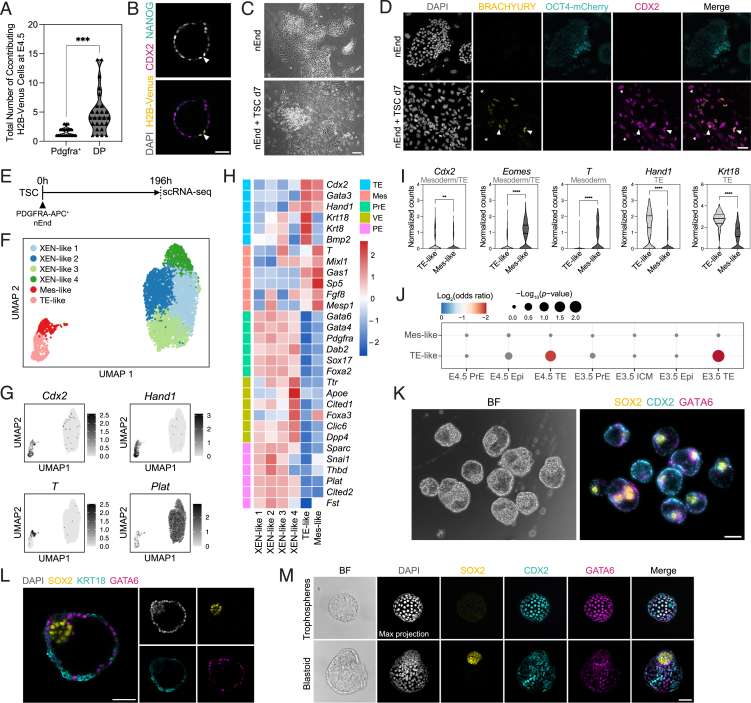
nEnd contributes to TE *in vivo* and differentiates into a TE-like cell types *in vitro*, related to Figure 3 (A) Total number of OCT4-mCherry-H2B-Venus DP and Pdgfra^+^ cells contributing to each embryo at E4.5 (DP: *n* = 36 embryos; Pdgfra^+^: *n* = 44 embryos). (B) Immunostaining of an OCT4-mCherry-H2B-Venus DP nEnd chimera embryo at E4.5 for indicated markers. (C) Bright-field images of nEnd and nEnd cultured in TSC medium. (D) Immunostaining of OCT4-mCherry nEnd and nEnd cultured in TSC medium for indicated markers. White arrowheads point to BRACHYURY/CDX2 co-expressing cells and white asterisk points to CDX2 single-positive cells. (E) Differentiation of nEnd in TSC medium followed by whole transcriptome analysis by scRNA-seq. (F) UMAP dimensional embedding of 6,094 nEnd cells in TSC medium. Top: coloring based on culture condition defined in (C); bottom: coloring based on Louvain clustering. (G) UMAP dimensional embedding showing single-cell expression of indicated markers. (H) Heatmap of candidate lineage markers expressed in log_2_ normalized clustered data. Scaled by row. (I) Expression of indicated TE and mesoderm-specific markers in TE-like (*n* = 357 cells) and Mes-like (*n* = 221 cells) clusters. (J) Gene overlap analysis of TE-like and Mes-like clusters with scRNA-seq of the mouse preimplantation embryo.^53^ Gray points represent *p* > 0.05. (K) Immunostaining of DP nEnd-derived blastoids for indicated markers, imaged by widefield microscopy. (L) Bright-field and immunostaining of a DP nEnd-derived blastoid for indicated markers. (M) Immunostaining of DP nEnd-derived blastoids and trophospheres for indicated markers. Values for (H) and (I) in Table S2. *p* values determined by unpaired t test, and error bars represent ± SD. Scale bars: 100 μm in (C) and (K); 50 μm in (B), (D), (L), and (M).

To test the developmental competence of different nEnd subpopulations, we used OCT4-mCherry nEnd labeled with a constitutive lineage marker (OCT4-mCherry-H2B-Venus), reintroduced 3 DP, Pdgfra^+^ or Oct4^+^ cells into host 8-cell embryos, and allowed these to develop for 48 h until E4.5. DP cells contributed extensively to 77.8% of injected embryos compared with poor contribution in just 45.5% of embryos injected with Pdgfra^+^ cells (Figures 3I, 3J, and S5A). DP cells populated all three lineages, while Pdgfra^+^ cells were only found in the PrE (Figures 3I and 3J). Injection of Oct4^+^ cells produced similar rates of chimerism to naive ESCs,^23^^,^^30^^,^^48^ with 53.49% of injected embryos showing Epi contribution and 13.95% contributing to both Epi and PrE (Figures 3I and 3J). DP cells localized and appeared morphologically indistinguishable to TE but did not upregulate the TE determinant CDX2 and were therefore termed TE position cells^62^ (Figures 3J and S5B). To test the capacity of a single cell for multi-lineage differentiation, we injected single DP or Oct4^+^ cells to 8-cell host embryos, assessed contribution following *in vivo* development through E6.5, and found widespread colonization of both embryonic and extra-embryonic lineages (Figure 3K).

As TSCs have previously been derived from an extra-embryonic endoderm-like intermediate,^63^ we asked whether nEnd could differentiate into TSCs *in vitro*. PDGFRA-APC single positive nEnd were differentiated in TSC medium^33^ for 7 days. These cultures were morphologically heterogeneous, (Figure S5C) with two CDX2-positive populations—one co-expressing the mesoderm marker BRACHYURY (T) and the other expressing only CDX2 (Figure S5D). To distinguish TE from mesodermal cell types, we performed scRNA-seq on nEnd differentiated in TSC medium (Figure S5E). The dataset contained 6,094 cells, detecting 32,285 genes that, upon sub-clustering and dimensionality reduction, produced 6 clusters visualized by uniform manifold approximation projection (UMAP)^64^ (Figure S5F). The clusters were annotated based on marker expression as TE-like, mesoderm-like (Mes-like) and XEN-like (Figures S5G and S5H). Although both the TE-like and Mes-like clusters expressed *Cdx2* and *Gata3*, only Mes-like expressed the mesoderm-specific markers *T*, *Mixl1*, and *Mesp1*, with TE-like cells expressing significantly higher levels of the TE-specific markers *Hand1*, *Krt8*, and *Krt18* (Figures S5H and S5I; Table S2). XEN-like clusters 1–4 expressed high levels of pan-endodermal and PE markers, such as *Gata6*, *Plat*, and *Sparc*. The appearance of a XEN-like phenotype in TSC medium is not surprising due to many similarities between TSC and XEN medium (see STAR Methods). Differentially expressed genes (DEGs) between TE-like and Mes-like clusters to the Nowotschin et al. dataset^53^ revealed the TE-like population was similar to E3.5 and E4.5 TE, while the Mes-like population appeared unrelated to the *in vivo* cell types in this dataset (Figure S5J).

We then asked whether DP nEnd could generate a complete stem cell-based embryo model of the preimplantation blastocyst, referred to as a blastoid.^65^ We applied conditions shown to generate blastoids from ESCs^57^ to our DP nEnd isolated by FACS and seeded these in AggreWells (Figure 3L). After 5 days in culture, these formed cavitated structures containing all 3 expected cell types of the blastocyst (Figures 3M, S5K, and S5L). This includes a SOX2-positive Epi-like center with a delaminated GATA6-positive PrE layer abutted to a cavity surrounded by CDX2/KRT18-positive TE-like cells produced at a high efficiency (61.3%) (Figures 3N and S5M).

### JAK/STAT signaling supports plasticity in OCT4-expressing endoderm

**Figure 4:**
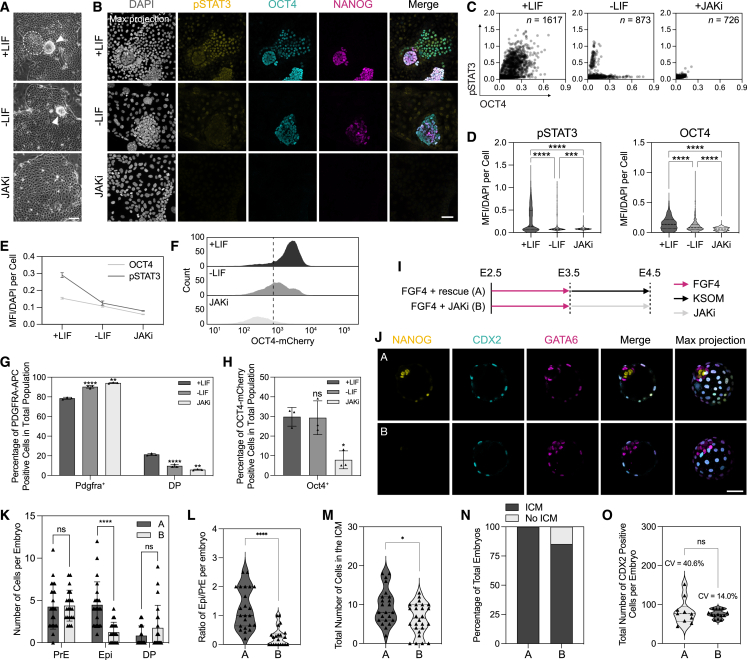
JAK/STAT signaling is required to maintain uncommitted PrE *in vitro* and *in vivo* (A) Bright-field images of OCT4-mCherry nEnd in control and treated conditions. Arrowheads indicate aggregates containing putative reverted cells and white dashed line highlights epithelial nEnd with compact morphology. (B) Immunostaining of OCT4-mCherry nEnd in control and treated conditions after 2 passages for indicated markers. (C) Single-cell quantification for pSTAT3 and OCT4 staining in (B) normalized to DAPI. *n* values indicate total number of cells quantified. (D) Quantification of median fluorescence intensity (MFI) normalized to DAPI for (B), where +LIF: *n* = 1,629 cells; −LIF: *n* = 873 cells; JAKi: *n* = 726 cells. (E) Total MFI normalized to DAPI for (B). (F) Flow cytometry histogram of OCT4-mCherry expression in nEnd for control and treated conditions. Dashed line indicates gating based on negative control. (G and H) Total expression by flow cytometry of G PDGFRA-APC and H OCT4-mCherry for control and treated conditions. Gating based on quadrants in Figure S3E. (I) Treatment regimen subjected to 8-cell embryos in (J)–(O). (J) Immunostaining of E4.5 embryos for indicated markers. Expression of GATA6 and NANOG was used for analysis performed in (K)–(N), and expression of CDX2 was used for analysis performed in (O). (K) ICM lineage allocation in treated embryos. (L) Ratio of Epi/PrE cells per embryo in treated embryos. (M) Total number of cells within the ICM of treated embryos. (N) Quantification of presence or absence of an ICM in treated embryos. (O) Total number of TE cells based on CDX2 expression in treated embryos (CV = coefficient of variance), where (A) *n* = 11 embryos; (B) *n* = 14 embryos. *n* values for treatment regimen outlined in (I) shown in (J)–(O) are (A) *n* = 23 and (B) *n* = 26 embryos. *p* values determined by unpaired t test, and error bars represent ± SD. Scale bars: 100 μM in (A); 50 μm in (B) and (J).

In addition to its role in ESC self-renewal,^66^^,^^67^^,^^68^ LIF-mediated JAK/STAT signaling supports PrE priming in ESCs and PrE progenitor states *in vivo*.^69^ We therefore assessed the influence of LIF withdrawal or culture in a JAK inhibitor (JAKi)^69^^,^^70^ on nEnd identity, which resulted in a loss of the DP population through a significant reduction in phosphorylated STAT3 (pSTAT3) and OCT4-mCherry expression (Figures 4A–4E). When blocking JAK/STAT activity, de-differentiation based on NANOG and OCT4 expression is lost (Figures 4B–4F), while flow cytometry revealed a significant increase in Pdgfra^+^ cells at the expense of the DP population (Figures 4G and 4H).

To discern the role of JAK/STAT signaling in PrE plasticity *in vivo*, we first cultured 8-cell embryos in FGF4 for 24 h to convert ICMs to PrE followed by transfer to either control media (FGF4 + rescue) or media containing JAKi (FGF4 + JAKi) for an additional 24 h (Figure 4I). Rescue embryos were able to correctly allocate both the Epi and PrE lineage as previously observed (Figures 1I–1L), while the JAKi-treated embryos contained only PrE or no ICM at all (Figures 4J–4N), with no significant difference in the number of TE cells (Figure 4O). This suggests that sustained JAK/STAT signaling is required to forestall PrE commitment, such that it retains competence for conversion toward Epi *in vivo* as well as *in vitro*.

### nEnd contains a subpopulation enriched for OCT4 targets

**Figure S6:**
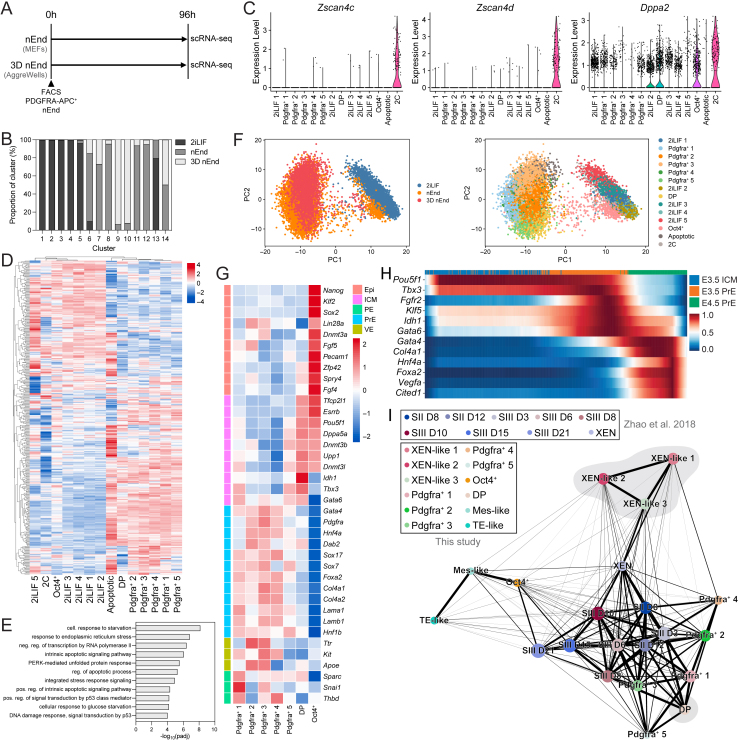
Clustering annotation strategy for 2iLIF, nEnd, and 3D nEnd scRNA-seq, related to Figure 5 (A) scRNA-seq strategy for nEnd and 3D nEnd. (B) Allocation of each culture condition per cluster in Figure 5A. (C) Differential expression of candidate 2C markers across all clusters. (D) Heatmap showing expression of genes related to apoptosis^80^ in log_2_ normalized clustered data. (E) GO analysis of selected biological processes of upregulated genes in the putative apoptotic cluster. (F) PCA of scRNA-seq dataset for 2iLIF, nEnd, and 3D nEnd. Left: coloring based on culture condition; right: coloring based on Louvain clustering. (G) Heatmap of candidate lineage markers in log_2_ normalized clustered data. Scaled by row. (H) Heatmap of scaled expression of candidate early (*Pou5f1*, *Tbx3*, *Fgfr2*, *Klf5*, *Idh1*, and *Gata6*) and late (*Gata4*, *Col4a1*, *Hnf4a*, *Foxa2*, *Vegfa*, and *Cited1*) PrE genes from scRNA-seq of ICM to PrE *in vivo* across pseudotime.^53^ Scaled by row. (I) PAGA of integrated *in vitro* nEnd, 3D nEnd and nEnd to TSC (this study), and somatic cell reprogramming to iPSCs from a XEN-like intermediate^77^ datasets using SCVI.^74^ Coloring based on Louvain clustering (XEN-like 1–3, Pdgfra^+^ 1–5, DP, Oct4^+^, Mes-like, and TE-like) or reprogramming stage (XEN, SII D8, SII D12, SIII D3, SIII D6, SIII D8, SIII D10, SIII D15, and SIII D21). Thicker lines indicate highly connected regions and thinner lines indicate regions with lower confidence. Values in Table S3.

**Figure 5:**
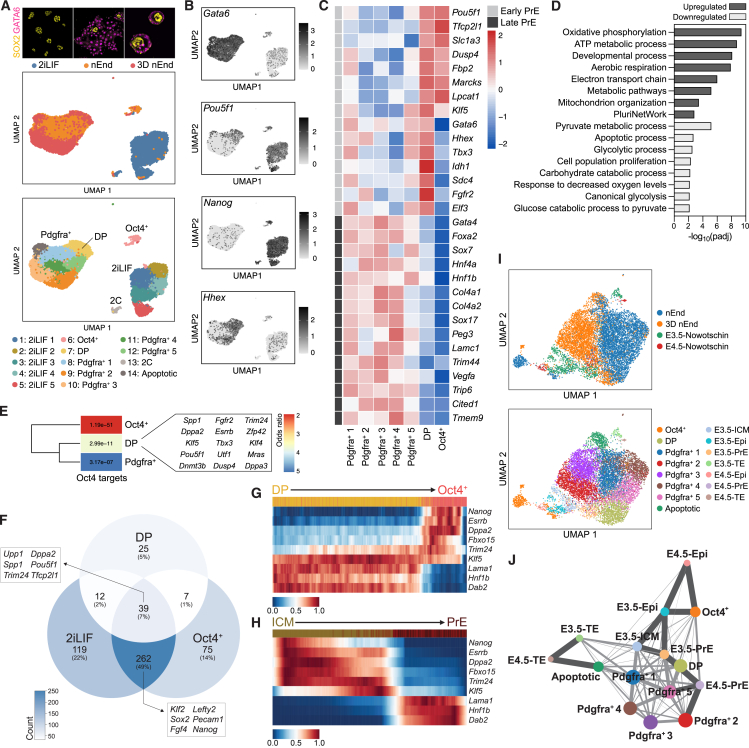
nEnd contains subpopulations mirroring stages of mouse preimplantation development (A) UMAP dimensional embedding of 14,788 2iLIF, nEnd, and 3D nEnd cells by scRNA-seq. Top: coloring based on culture condition shown above, including representative immunostaining for SOX2 and GATA6; bottom: coloring based on Louvain clustering. (B) UMAP dimensional embedding showing single-cell expression of indicated markers. (C) Heatmap of candidate lineage markers in log_2_ normalized clustered data for early and late PrE *in* vivo,^53^ defined in Figure S6H. Scaled by row. (D) GO analysis of selected biological processes of up- and downregulated genes in the DP cluster. (E) Gene overlap analysis of Oct4^+^, DP, and Pdgfra^+^ 1–5 clusters with 700 downstream targets of Oct4.^71^ Color scale is based on odds ratio and grids labeled with *p* value indicating significance of the respective odds ratio. (F) Venn diagram showing overlap of Oct4^+^, DP, and 2iLIF clusters based on differential upregulation of 1,361 pluripotency and Epi-specific genes.^22^^,^^53^^,^^72^^,^^73^ (G and H) Heatmap of scaled expression of indicated genes from scRNA-seq of (G) DP and Oct4^+^ nEnd clusters *in vitro* and (H) ICM to PrE *in vivo*^53^ across latent time. Top bar shows progression from (G) DP (yellow) to Oct4^+^ (pink) and (H) E3.5 ICM (ochre) to E4.5 (burgundy). (I) UMAP dimensional embedding of integrated *in vitro* nEnd and 3D nEnd with *in vivo* data^53^ using scVI.^74^ Top: coloring based on culture condition (nEnd, 3D nEnd) or developmental stage (E3.5, E4.5). Bottom: coloring based on Louvain clustering. (J) PAGA of integrated scRNA-seq dataset shown in (I). Dark gray lines: highly connected regions; light gray lines: regions with lower confidence. Values in Table S3. See also Figures S6 and S7.

To construct a transcriptional map of nEnd heterogeneity, we performed scRNA-seq of nEnd in adherent culture and 3D nEnd in AggreWells compared with 2iLIF ESCs (Figure S6A). Quality control produced a dataset comprised of 14,788 cells and 32,285 genes resolving into 14 clusters (Figures 5A and S6B). 2iLIF ESCs produced 5 clusters and the majority of a small cluster annotated as 2C based on expression of 2C-like genes^26^ (Figure S6C). nEnd and 3D nEnd clusters expressed *Pdgfra* and *Gata6*, except the de-differentiated cluster 6 expressing pluripotency markers, including *Nanog* and *Oct4*, annotated as Oct4^+^ (Figure 5A, 5B). Within the *Pdgfra*/*Gata6*-expressing clusters, *Oct4* expression was found in cluster 7 and 14. As cluster 14 expressed high levels of apoptotic markers (Figures S6D and S6E), we refer to this as apoptotic and excluded it from further analysis. Cluster 7 was annotated as DP and the remaining nEnd and 3D nEnd clusters as Pdgfra^+^ 1–5 (Figure 5A). Principal-component analysis (PCA) showed that the first principal component (PC1) separated 2iLIF ESCs and a subset of nEnd and 3D nEnd cells from the bulk endoderm populations (Figure S6F). The Oct4^+^ cluster bridges the pluripotent 2iLIF clusters to the endodermal clusters, with the DP cluster being an anchoring point for the latter.

We found Epi-related genes specifically upregulated in the Oct4^+^ cluster, and PrE, VE, and PE markers heterogeneously expressed in Pdgfra^+^ 1–5 (Figure S6G). The DP cluster expressed canonical PrE genes while maintaining modest levels of select ICM and pluripotency genes. When expanding the panel of PrE markers to those specific to early and late PrE *in vivo*^53^ (Figures 5C and S6H), the nEnd clusters partitions into two, where DP resemble early PrE and the remaining nEnd clusters late PrE. As XEN cells have been identified as an intermediate in the reprogramming of somatic cells to pluripotency,^75^^,^^76^ we considered whether nEnd and XEN intermediates represent a common cell type. We exploited scRNA-seq of chemical reprogramming toward iPSCs^77^ and integrated our datasets using scVI.^74^ We quantified cell type connectivity with partition-based graph abstraction (PAGA)^78^ (Figure S6I) and found that the biproduct XEN-like population derived from TSC differentiation of nEnd (Figure S5) strongly aligns with the XEN-like intermediates identified in reprogramming, while the nEnd clusters closer resemble the cell types arising later in the trajectory toward pluripotency.

**Figure S7:**
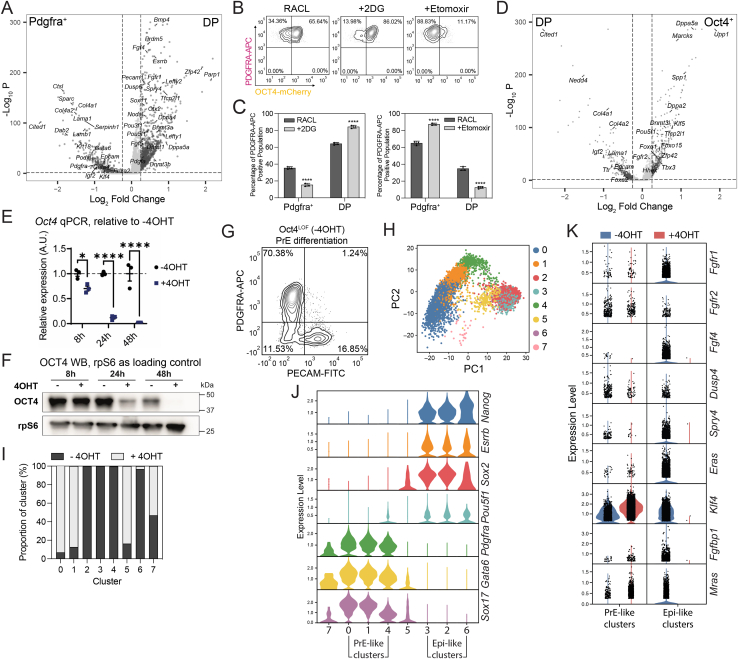
Gene expression characterization and validation in OCT4-mCherry and Oct4^LOF^ nEnd scRNA-seq datasets, related to Figures 5 and 6 (A) Volcano plot of DEGs between DP and Pdgfra^+^ clusters representative of log_2_ fold change > 0.25 and *p* < 0.05. (B) Flow cytometry contour plots gated for PDGFRA-APC expression of OCT4-mCherry nEnd cultured in 2DG or etomoxir for 5 days. (C) Quantification of PDGFRA-APC expression in nEnd treated with 2DG or etomoxir compared with control. (D) Volcano plot of DEGs between Oct4^+^ and DP clusters representative of log_2_ fold change > 0.25 and *p* < 0.05. (E and F) (E) RT-qPCR and (F) western blot of Oct4^LOF^ ESCs cultured in 2iLIF for *Oct4*/OCT4 ± 4OHT at indicated time intervals. rpS6 used as a loading control in (F). (G) Flow cytometry contour plot of Oct4^LOF^ PrE following 7 days of differentiation in RACL. (H) PCA of scRNA-seq dataset for Oct4^LOF^ nEnd ± 4OHT at 120 h, where coloring is based on Louvain clustering. (I) Allocation of each culture condition per cluster in (H). (J) Violin plots showing expression of indicated markers across Louvain clusters in scRNA-seq dataset in (H). (K) Violin plots showing expression of select genes related to FGF/ERK signaling^80^ from Figure 6G across PrE-like and Epi-like clusters defined in (J). *p* values determined by unpaired t test, and error bars represent ± SD.

GO analysis of the top 300 DEGs between DP and Pdgfra^+^ 1–5 clusters (Figure S7A) revealed an enrichment of metabolic terms associated with oxidative phosphorylation, mitochondrial activity, and pluripotency in DP cells, while Pdgfra^+^ cells were associated with glycolysis (Figure 5D). To verify these observations *in vitro*, we cultured OCT4-mCherry nEnd sorted for PDGFRA-APC expression in either an inhibitor of glucose metabolism (2-deoxyglucose (2DG)) or an inhibitor of fatty acid oxidation (etomoxir), where 2DG resulted in a significant increase in DP cells, while etomoxir led to an increase in Pdgfra^+^ cells (Figures S7B and S7C).

As many DEGs in the DP cluster are known interactors of OCT4,^71^^,^^79^ we compared all differentially upregulated genes in Oct4^+^, DP, and Pdgfra^+^ 1–5 clusters to 700 functional downstream target genes of OCT4^71^ (Figure 5E). The DP cluster positioned itself as an intermediate between Pdgfra^+^ 1–5 and Oct4^+^, where OCT4 targets were modestly enriched in the DP cluster and strongly enriched in the Oct4^+^ cluster. The transcriptional changes occurring between the DP and Oct4^+^ clusters are consistent with an endodermal cell type progressing toward an Epi-like state, with upregulation of genes related to pluripotency and Epi, and downregulation of endoderm and extra-cellular matrix markers (Figure S7D). When comparing differentially upregulated genes across DP, Oct4^+^, and 2iLIF clusters, we observed a large gene overlap between Oct4^+^ and 2iLIF cells (49% of DEGs), including *Nanog*, *Sox2*, and *Klf2*, and a modest subset of genes shared by all three conditions (7% of DEGs), including *Pou5f1*, *Tfcp2l1*, and *Trim24*, all of which are related to pluripotency and known OCT4 targets^71^ (Figure 5F).

RNA velocity^54^^,^^55^ was used to determine latent time and infer directionality to the PrE-to-Epi transition. We compared the single-cell trajectory of gene expression from the DP to Oct4^+^ clusters to the transition from E3.5 ICM to E4.5 PrE^53^^,^^56^ for select downstream targets of OCT4^71^ (Figures 5G and 5H). Here, the temporal sequence of gene expression was inversely related, with early PrE markers, such as *Dab2* and *Klf5*, being downregulated and ICM/Epi markers, such as *Dppa2* and *Esrrb*, being upregulated during the transition from DP to Oct4^+^.

As DP nEnd shares many characteristics with early PrE just prior to commitment, we compared this cluster to PrE specification *in vivo* by integrating our nEnd scRNA-seq dataset with mouse preimplantation embryo stages E3.5 and E4.5^53^ using scVI^74^ (Figure 5I). Using PAGA,^78^ we found our DP cluster positioned directly between E3.5 and E4.5 PrE, while our Oct4^+^ cluster positioned between E3.5 and E4.5 Epi (Figure 5J).

### OCT4 supports plasticity with ESRRB required for PrE-to-Epi transitions

**Figure 6:**
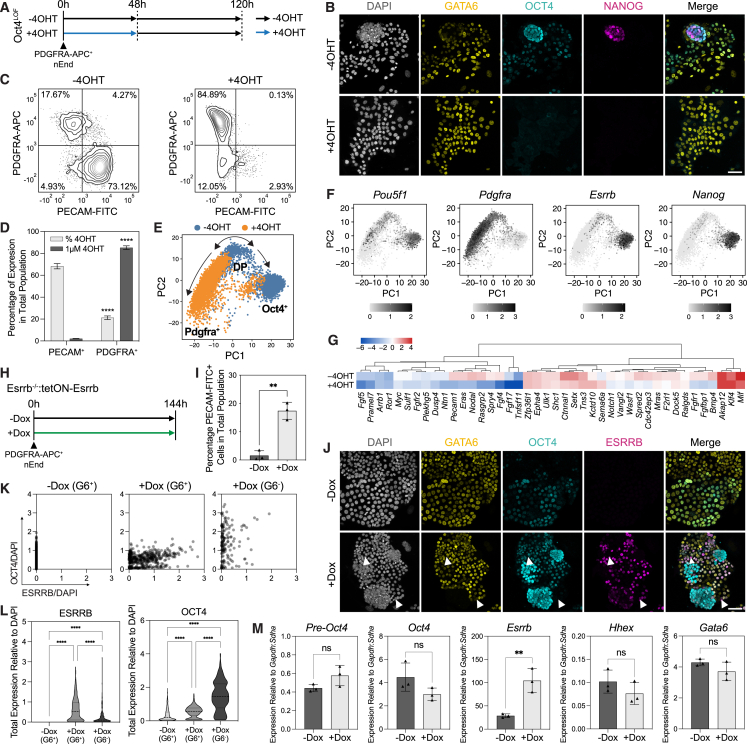
OCT4 regulates nEnd plasticity and ESRRB is a gatekeeper of nEnd de-differentiation (A) *Oct4* depletion in Oct4^LOF^ nEnd performed for (B)–(G). PDGFRA-APC^+^ nEnd isolated by FACS and cultured +4OHT (−Oct4) or −4OHT (+Oct4) for 48 h, followed by an additional 72 h in normal RACL medium. (B) Immunostaining of Oct4^LOF^ nEnd ± 4OHT at 120 h for indicated markers. (C) Flow cytometry contour plots of Oct4^LOF^ nEnd −4OHT (left) and +4OHT (right) stained for PECAM-FITC and PDGFRA-APC. Quadrants: gating based on a negative control. (D) Quantification of proportions of PECAM-FITC^+^ and PDGFRA-APC^+^ cells based on total expression by flow cytometry in (C). (E) PCA of scRNA-seq dataset for Oct4^LOF^ nEnd ± 4OHT at 120 h. Coloring indicates treatment and black arrows indicate inferred trajectories of dataset variance. (F) UMAP dimensional embedding showing single-cell expression of indicated markers. (G) Heatmap of candidate lineage markers in log_2_ normalized clustered data for FGF/ERK-related genes.^80^ Scaled by row. (H) Esrrb induction with EKOiE nEnd. PDGFRA-APC^+^ EKOiE nEnd isolated by FACS and cultured in +Dox (+Esrrb) or −Dox (−Esrrb) for 7 days. (I) Quantification of total expression of PECAM-FITC in EKOiE nEnd −Dox. (J) Immunostaining of EKOiE nEnd ±Dox for indicated markers. (K and L) Quantification of (J) for OCT4 and ESRRB in cells grouped by GATA6 expression for (K) single cell and (L) total expression. (M) RT-qPCR of PDGFRA-APC^+^ EKOiE cells ±Dox isolated by FACS for indicated markers. *p* values determined by unpaired t test, and error bars represent ± SD. Scale bars, 50 μm. See also Figures S7.

To establish whether Oct4 is directly regulating plasticity in nEnd or merely a marker of early PrE, we generated nEnd from homozygous 4OHT-inducible Oct4 loss-of-function mouse ESCs (Oct4^LOF^)^19^ (Figures S7E–S7G). We isolated PDGFRA-APC-positive cells by FACS and cultured these with 4OHT to induce *Oct4* deletion (Figure 6A). This resulted in a loss of OCT4 protein (Figure 6B) and loss of de-differentiated Epi-like cells alongside an increase in the Pdgfra^+^ and DN populations (Figures 6C and 6D). We then used scRNA-seq of Oct4^LOF^ nEnd to characterize the transcriptional response to Oct4 depletion. PCA revealed a primary separation of the dataset based on the trajectory from DP to either Pdgfra^+^ or Oct4^+^ nEnd (Figures 6E and 6F). We found that untreated nEnd (−4OHT) contained all three expected populations, while the cells from 4OHT-mediated Oct4 depletion (+4OHT) were primarily Pdgfra^+^ cells and missing the DP and Oct4^+^ populations (Figures 6E, 6F, S7H–S7J). As Oct4 is known to regulate FGF/ERK signaling,^15^^,^^18^^,^^52^^,^^68^ we asked whether Oct4 supports this pathway in nEnd by comparing DEGs between −4OHT and +4OHT to genes associated with the FGF/ERK pathway and only observed reduced expression of those related to Epi identity (Figure 6G). We then further partitioned the dataset into PrE-like and Epi-like clusters based on lineage-specific markers (Figure S7J) and found that Oct4-dependent FGF/ERK signaling components were specifically downregulated within Epi-like clusters (Figure S7K).

We recently found that Esrrb safeguards the decision point between Epi- and PrE-like cells to support plasticity.^56^^,^^81^ We probed the relationship between Oct4 and Esrrb during de-differentiation from DP to an Oct4^+^ state by employing doxycycline (Dox)-inducible Esrrb knockout (Esrrb^−/−^:tetON-Esrrb [EKOiE]) ESCs.^82^ These were differentiated to PrE by inducing Esrrb expression for the first 48 h, after which Dox was removed for the duration of differentiation^56^ and subsequent nEnd expansion. EKOiE nEnd was sorted based on PDGFRA-APC expression by FACS and seeded in the presence (+Dox) or absence (−Dox) of Dox for 7 days (Figure 6H). Only +Dox nEnd was able to undergo de-differentiation (Figures 6I and 6J), while −Dox nEnd formed large epithelial sheets with extensive co-expression of OCT4 and GATA6 but lacking ESRRB expression (Figures 6J and 6K). As OCT4 protein is significantly increased in GATA6^+^ nEnd +Dox compared with −Dox (Figure 6L), we asked whether ESRRB regulates *Oct4* transcription. RT-qPCR of nEnd +Dox and −Dox revealed that levels of *Oct4* and nascent *Oct4* were unchanged despite increased levels of *Esrrb* (Figure 6M). This suggests that ESRRB, while not required for DP nEnd identity, is crucial for activation of the Epi-related gene regulatory network during the de-differentiation event.

### The DP nEnd enhancer landscape supports plasticity

**Figure S8:**
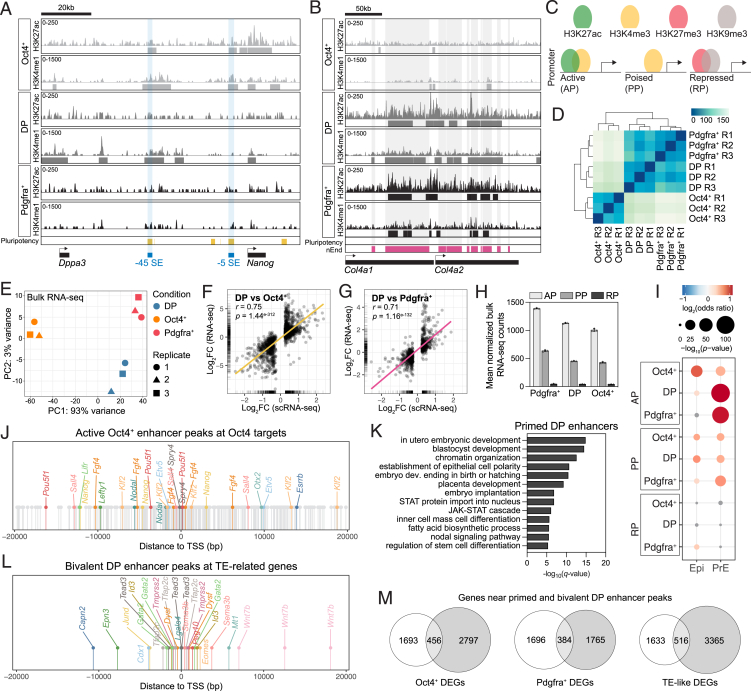
Chromatin states in nEnd subpopulations demonstrate specific developmental characteristics, related to Figure 7 (A) Profiles of H3K4me1 and H3K27ac for Oct4^+^, DP, and Pdgfra^+^ nEnd upstream of *Nanog* compared with defined pluripotency (yellow) enhancers,^48^ highlighted in gray with −5 and −45 Nanog SE annotated in blue. Bigwigs generated from 3 biological replicates. (B) Profiles of H3K4me1 and H3K27ac for Oct4^+^, DP, and Pdgfra^+^ nEnd at *Col4a1* and *Col4a2* loci compared with defined pluripotency (yellow) and nEnd (magenta) enhancers.^48^ Bigwigs generated from 3 biological replicates. (C) Histone mark categories for promoter states. Active: H3K27ac and H3K4me1; poised: H3K27me3 only; repressed: H3K27me3 and H3K9me3. (D) Unbiased hierarchical clustering of bulk RNA-seq dataset for Oct4^+^, DP, and Pdgfra^+^ nEnd biological replicates. (E) PCA of bulk RNA-seq dataset for Oct4^+^, DP, and Pdgfra^+^ nEnd replicates. (F and G) Scatterplots showing correlation between DEGs (log_2_ fold change > 1.5; adjusted *p* < 0.05) in bulk RNA-seq compared with scRNA-seq for (F) DP vs. Oct4^+^ nEnd and (G) DP vs. Pdgfra^+^ nEnd. (H) Quantification of expression for mean normalized bulk RNA-seq counts in DP, Oct4^+^, and Pdgfra^+^ nEnd across promoter categories outlined in (C). (I) Gene overlap analysis of promoter peak categories defined in (C) annotated to the nearest TSS against DEGs from scRNA-seq data of the mouse preimplantation Epi and PrE *in vivo*.^72^ Gray points represent *p* > 0.05. (J) Position of active Oct4^+^ peaks annotated to the nearest TSS (±20 kb) relative to genes selected for Figure 7F and other downstream targets of Oct4.^71^ (K) GO analysis of selected biological processes for primed DP peaks. (L) Euler diagrams showing overlap between annotated genes within ±20 kb of primed and bivalent DP peaks and DEGs in scRNA-seq for Oct4^+^, Pdgfra^+^, and TE-like clusters to DP nEnd. (M) Position of bivalent DP peaks annotated to the nearest TSS (±20 kb) relative to selected genes related to TE fate. Values in Tables S4 and S5.

**Figure 7:**
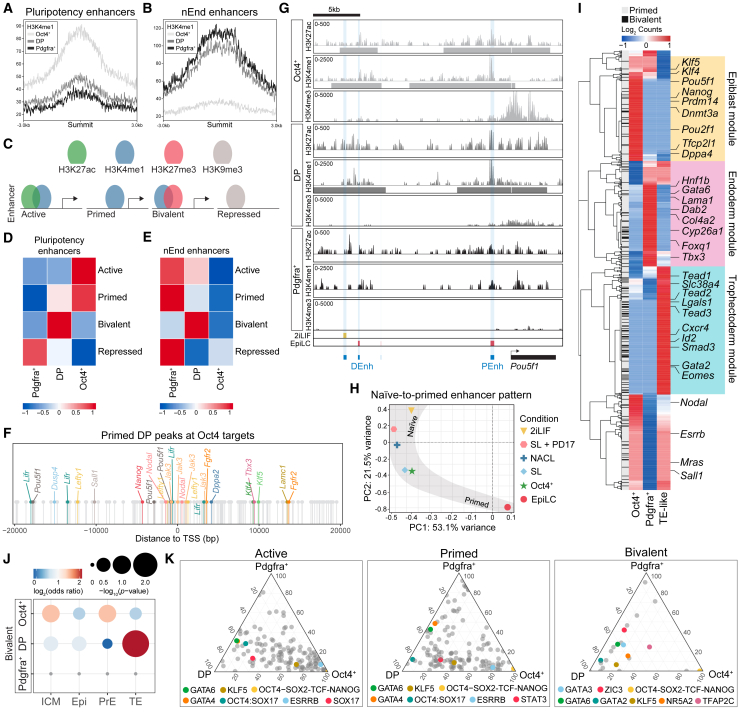
Status of lineage-specific regulatory elements supports DP nEnd plasticity and predicts future differentiation competence (A and B) H3K4me1 occupancy at defined (A) pluripotency and (B) nEnd enhancers^48^ in Oct4^+^, DP, and Pdgfra^+^ nEnd. (C) Histone mark categories for enhancer states. Active: H3K27ac and H3K4me1; primed: H3K4me1; bivalent: H3K4me1 and H3K27me3; repressed: H3K9me3. (D and E) Heatmap of log_2_ normalized peaks overlapping with annotated (D) pluripotency and (E) nEnd enhancers. Scaled by row. (F) Position of DP-primed peaks annotated to the nearest TSS (±20 kb) of select downstream target genes of Oct4.^71^ (G) Profiles of H3K4me1, H3K27ac, and H3K4me3 for Oct4^+^, DP, and Pdgfra^+^ nEnd upstream *Oct4* compared with defined 2iLIF (yellow) and EpiLC (magenta) enhancers.^89^ The proximal enhancer (PEnh) and distal enhancer (DEnh) of *Oct4* are highlighted in blue. Bigwigs generated from 3 biological replicates. (H) PCA of global naive (2iLIF) to primed (EpiLC) enhancer patterns^89^ across indicated conditions. (I) Heatmap of scRNA-seq data from log_2_ normalized DEGs in Oct4^+^, Pdgfra^+^, and TE-like clusters compared with genes with a TSS ±20 kb of primed and bivalent DP peaks. Annotations include lineage-specific genes for Epi, endoderm, and TE. (J) Gene overlap analysis of bivalent enhancer peaks annotated to the nearest TSS against DEGs from scRNA-seq data of the mouse preimplantation embryo *in vivo*.^90^ Gray points: *p* > 0.05. (K) Ternary plots of motif enrichment across Oct4^+^, DP, and Pdgfra^+^ nEnd in the active, primed, and bivalent categories. *p* < 0.05 for at least one population for all motifs, axis represents −log_10_(*p* value). Gray dots indicate all motifs and colored dots highlight motifs of interest. Values in Table S4. See also Figure S8.

To understand transcriptional plasticity in nEnd, we profiled chromatin in regulatory regions using cleavage under targets and tagmentation (CUT&Tag)^83^ across nEnd subpopulations focusing on key regulatory histone modifications: H3K27ac and H3K4me1 for active and primed histone marks, respectively,^84^ H3K27me3 for polycomb repressive complex 2 activity and enhancer priming,^85^ and H3K9me3 for repression.^86^ To annotate consensus peaks within our dataset, we incorporated enhancer peak sets for either pluripotency or nEnd.^48^ Pluripotency genes, such as *Nanog*, were marked by both H3K27ac and H3K4me1 in Oct4^+^ nEnd, by H3K4me1 only in DP nEnd, and by neither in Pdgfra^+^ nEnd (Figure S8A). This contrasts endodermal loci, such as *Col4a1* and *Col4a2*, which were enriched in H3K27ac and H3K4me1 at nEnd enhancers in DP and Pdgfra^+^ nEnd, but not in Oct4^+^ cells (Figure S8B). Global levels of H3K4me1 at pluripotency and nEnd enhancers across all three populations were enriched at pluripotency enhancers in Oct4^+^ cells and at nEnd enhancers in Pdgfra^+^ cells, with partial enrichment for both enhancer classes in DP cells (Figures 7A and 7B). Although the deposition of H3K4me1 characterizes priming of enhancer elements, bivalent regulatory regions with co-binding of histone marks such as H3K4me1 and H3K27me3 are also associated with both active and repressive transcriptional outcomes.^87^ To address how enhancer states in DP nEnd could account for plasticity, we focused on *de novo* enhancers defined by the presence of different histone posttranslational modifications (PTMs) outside of annotated promoters.^88^ These regions were classified based on H3K4me1 together with H3K27ac (active), H3K4me1 alone (primed), H3K4me1 together with H3K27me3 (bivalent), and H3K9me3 (repressed) (Figure 7C). When comparing our pluripotency enhancer set^48^ across nEnd subpopulations, we found that these were active or primed in Oct4^+^ cells, primed or bivalent in the DP cells, and repressed in Pdgfra^+^ nEnd (Figure 7D). This contrasts nEnd enhancers, which were primarily active or primed in Pdgfra^+^ nEnd but bivalent in DP nEnd and inactive in Oct4^+^ cells (Figure 7E).

Because enhancer activity can be difficult to correlate with gene expression, we also determined the promoter status across DP, Pdgfra^+^, and Oct4^+^ nEnd based on CUT&Tag for H3K4me3, a histone mark associated with promoters of actively transcribed genes.^91^^,^^92^ Promoters were categorized based on the presence of both H3K4me3 and H3K27ac (active promoters [APs]), H3K4me3 alone (poised promoters [PPs]), and H3K27me3 together with H3K9me3 (repressed promoters [RPs])^88^ within annotated promoter regions (Figure S8C). As our scRNA-seq clustering in Figure 5 was based on *in silico* annotation, we performed bulk RNA sequencing (RNA-seq) on individual DP, Pdgfra^+^, and Oct4^+^ nEnd (Figures S8D and S8E) and found that these highly correlate with scRNA-seq gene expression (Figures S8F and S8G). The RNA-seq dataset also validated our promoter classification, with promoters defined as active associated with genes undergoing high levels of transcription (Figure S8H). When comparing our promoter peak sets to DEGs from scRNA-seq data of the mouse preimplantation Epi and PrE *in vivo*,^72^ genes regulated by DP and Pdgfra^+^ APs overlapped with PrE-specific genes, while genes regulated by Oct4^+^ APs were enriched for Epi-specific genes (Figure S8I).

To relate enhancer regulatory status to transcriptional plasticity, we annotated peaks to their nearest transcription start site (TSS). A conservative estimate of the genes regulated by primed DP enhancers based on a proximity of ±20 kb produces a list of numerous key OCT4 targets,^71^ including *Dppa3*, *Tfcp2l1*, *Esrrb*, and *Oct4* itself (Figure 7F), where priming corresponds to activation in de-differentiated Oct4^+^ cells (Figure S8J). Although Nanog is primed in DP nEnd only at its −45 kb super enhancer (SE), both the −45 and −5 kb SEs are activated in Oct4^+^ cells, where the −45 kb SE also regulates nearby *Dppa3*^93^^,^^94^ (Figure S8A). GO analysis of genes adjacent to primed DP peaks revealed enrichment in blastocyst development, JAK/STAT signaling, and ICM differentiation (Figure S8K).

During the naive-to-primed pluripotency transition, there is a global rewiring of the chromatin enhancer landscape where genes differentially utilize their proximal or distal enhancers to regulate gene expression.^89^^,^^95^^,^^96^ The *Oct4* locus employs its distal enhancer to regulate expression in the naive 2iLIF ESCs, then engages its proximal enhancer in metastable S/L ESCs, followed by a complete switch to proximal enhancer activity in primed Epi-like cells (EpiLCs).^96^ Using defined naive and primed enhancer annotations,^89^ we looked at the chromatin near the *Oct4* locus. We found activation of both 2iLIF and EpiLC enhancers in Oct4^+^ nEnd but only priming at these regions in DP nEnd, while Pdgfra^+^ lacked peaks at both enhancer regions as well as promoter activation (Figure 7G). As neither the proximal nor distal enhancer of *Oct4* appears to be acetylated in the DP nEnd, despite modest levels of transcription based on H3K4me3 at the promoter and within the gene body,^91^^,^^97^ this suggests the involvement of an additional *Oct4* enhancer element in early endoderm.

To compare active enhancers in the Oct4^+^ population to different pluripotent states marked by H3K27ac, we used naive ESCs in 2iLIF,^89^ S/L supplemented with PD17^81^, and N2B27 supplemented with Activin A, CHIR99021 and LIF (NACL),^36^ metastable naive S/L ESCs, and primed EpiLCs.^89^ Comparing the distribution of H3K27ac across the genome in each of these states creates a continuum visualized by PCA from which the pattern of naive-to-primed enhancer activation can be traced. Here, Oct4^+^ nEnd positions as an intermediate along this trajectory together with S/L ESCs (Figure 7H), pointing to a chromatin state resembling the peri-implantation Epi.

How do these plastic enhancer states relate to lineage-specific re-engagement during nEnd de-differentiation toward TE? Although active DP enhancers are found near endoderm genes and primed DP enhancers near Epi genes, we found that bivalent DP enhancers were in proximity to TE determinants such as *Tfap2c*, *Eomes*, and *Gata2* (Figure S8L). To extend this analysis, primed and bivalent regulatory elements in DP nEnd were used to predict the lineage trajectory through activation of lineage-specific genes in transcriptomes for Epi, committed endoderm and TE-like clusters (Figures 7I and S8M). Additionally, bivalent DP enhancers specifically regulate genes overlapping with the preimplantation TE *in vivo* (Figure 7J).

To explore the factors responsible for the DP, Oct4^+^, and Pdgfra^+^ nEnd enhancer landscape, we performed motif analysis on their enhancer sets. The OCT4:SOX17 motif was present in active and primed DP nEnd, but not the OCT4-SOX2-TCF-NANOG motif, which was only present in Oct4^+^ cells, where neither was found in Pdgfra^+^ (Figure 7K). The STAT3 motif was similarly only present in DP nEnd, further supporting the role of JAK/STAT signaling in this cell type. Bivalent DP nEnd contained TE-specific motifs, such as GATA2, GATA3, and TFAP2C, consistent with the potential of DP nEnd to undergo TE differentiation. Taken together, our findings suggest a phenomenon where the persistence of a particular set of lineage-specific TFs maintain the early extra-embryonic endoderm in a permissive state for multi-lineage differentiation.

## Discussion

In this paper, we demonstrated that the early PrE is sufficient to build a mouse and we provide molecular insight into pioneering work on the regulative nature of early mammalian development.^4^ The capacity of the PrE to regenerate all cell types of the blastocyst is governed by the status of lineage-specific enhancers and persistence of key TFs. Despite the onset of differentiation driven by active endoderm enhancers, competence for Epi and TE re-specification is supported by primed and bivalent enhancers, respectively. The maintenance of these three enhancer states in a single cell type sustains a multi-lineage potential approaching totipotency. OCT4 is key to this process as a factor that sits at the pinnacle of the gene regulatory network governing plasticity in addition to its well-known role in supporting pluripotency.

The amount of OCT4 expressed determines cell identity, where in ESCs, high and low levels of OCT4 enable differentiation toward endoderm and mesoderm or TE, respectively, while moderate levels promote pluripotency^52^^,^^98^ and sustain enhancer pliability.^99^ In eutherian mammals, it is a single gene, *Pou5f1*, which supports this functional diversity,^100^ and it is not surprising that *Oct4* is extensively conserved in evolution, where it can regulate differentiation competence via two paralogs, POU5F1 and POU5F3.^101^^,^^102^^,^^103^^,^^104^ Does OCT4 support these functions by preparing enhancers to respond to signaling? OCT4 binds enhancers associated with signaling-dependent genes, including response to TGF-β and ERK,^81^^,^^105^ and exhibits pioneer-like activity during reprogramming.^106^ Perhaps, as we describe here, *Oct4* functions as a plasticity TF, priming enhancers for lineage-specific responses to signaling, functioning as more of an enabler than a direct driver of transcription.

If OCT4 is a passive player supporting plasticity, then what signals sustain these responsive enhancer states? Expansion of the DP population and subsequent PrE-to-Epi transition *in vitro* and *in vivo* requires JAK/STAT signaling. As LIF signaling is associated with diapause, where differentiation is indefinitely stalled^107^ and overexpression of pSTAT3 allows indefinite propagation of morula-like cells,^108^ the role of this pathway in the DP population may be to block commitment of a plastic cell type. This is consistent with dependence of DP nEnd on oxidative phosphorylation, which, in turn, is linked to enhanced diapause via suppression of glycolysis.^109^

Although sustained FGF/ERK signaling is thought to drive PrE commitment,^39^ here, the duration of FGF4 treatment is shorter, performed at an earlier development time enabling rapid PrE maturation, and followed by a longer length of time for regeneration. Moreover, JAK/STAT signaling preserves plasticity and allows reactivation of Epi and TE networks during aberrant development. We also find Esrrb as a competence factor for both Epi and PrE,^56^ consistent with its requirement in ESCs to maintain plasticity and block commitment in response to ERK induction.^81^

While ESCs are generally considered closest to blastocyst-stage Epi, certain rare populations maintain the capacity for PrE and TE differentiation.^23^^,^^48^ Although not canonically defined as totipotent as these cells cannot generate an embryo, we previously referred to these as experimentally totipotent as they co-express certain PrE and Epi determinants, priming them for multi-lineage differentiation.^30^ The presence of a similar subpopulation in nEnd suggests that *in vitro* culture traps developmental transition states that may be relatively fleeting *in vivo*, where DP nEnd can be repeatedly isolated and used as a model closer to canonical totipotency than that found in pluripotent culture.

What are the trajectories of cell state transitions during reprogramming and differentiation, and how do these relate to Oct4? The emergence of a XEN-like intermediate during conversion of somatic cells to iPSCs, either en route to pluripotency^76^^,^^110^^,^^111^ or, alternatively, as a by-product of reprogramming,^75^ were initially reminiscent of nEnd. However, our analysis of transcriptomic data demonstrates that these XEN-like intermediates resemble postimplantation-stage endoderm or XEN cells and that nEnd subpopulations arise much closer to pluripotency. Moreover, our data suggest that the transition between DP and Oct4^+^ nEnd does not occur via a 2C-like intermediate but rather as a direct conversion between lineages.^63^^,^^112^

Although the TE is an innovation of eutherian mammals, the PrE/hypoblast and its descendants originate deep in amniote evolution. The PrE is associated with yolk sac formation, anterior-posterior axis formation, and positioning of the starting point for gastrulation.^113^^,^^114^ But why does the early PrE maintain a plastic population in preimplantation development? Because high-grade human embryos and successful implantation correlates with PrE quality,^115^ we suggest that the embryo needs this reservoir as a developmental insurance policy in case of aberrant development. Following placental evolution, the yolk sac begins to become somewhat redundant, but as we find its founder cells are required for regenerative purposes in preimplantation development, this could explain its conservation. Unlike the TE, where aneuploidy is common, the PrE is composed of normal cells that actively participate in elements of embryonic gut development.^50^^,^^53^ These cells therefore represent an ideal source of replacement cells to compensate for damage during preimplantation development.

### Limitations of the study

Although we found that the PrE *in vivo* is capable of regenerating an intact embryo and nEnd can generate blastoids *in vitro*, we have yet to demonstrate that they can undergo further development^116^ and/or maintain potential without numerous small molecules and growth factors in the blastoid media. Further lineage tracing is also required to definitively demonstrate PrE contribution to later stages of embryonic development and adulthood in regeneration. In both blastoid and TSC media, nEnd differentiates to Cdx2-expressing TE-like cells, but when injected into host embryos, nEnd integrating within the TE never expresses CDX2. Is this because they never become true TE or because these cells take time to adopt the TE program? ESCs that contribute to the TE following implantation also adopt this TE position phenotype,^62^ suggesting that overwriting pluripotency to drive TE fate takes time. Perhaps the answers to these questions will become apparent as more mechanistic studies become possible *in vivo*, replacing nEnd as an *in vitro* proxy for the preimplantation PrE.

Although the notion that the PrE provides a supportive reservoir for development could have profound implications, we have only identified the plasticity of this cell type in one species and identified two key TFs, Oct4 and Esrrb, whose persistence is critical. How many TFs are required and what defines their threshold concentration are questions that remain unanswered. Moreover, if plasticity is a function of persistence and dilution, how is it maintained *in vitro* in an indefinitely proliferating nEnd population and why do these cells continue to express Oct4?

## STAR★Methods

### Key resources table

**Table:** 

REAGENT or RESOURCE	SOURCE	IDENTIFIER
**Antibodies**		

See Table S6 for antibodies used in this study	N/A	N/A

**Chemicals, peptides, and recombinant proteins**		

Leukemia inhibitory factor (LIF)	Made in house	N/A
CHIR99021 (GSKβ inhibitor)	Axon Medchem	Cat#1386
PD0325901 (PD03, MEK inhibitor)	Sigma Aldrich	Cat#PZ0162
Activin A	Peprotech	Cat#120-14E
Doxycycline (Dox)	Sigma Aldrich	Cat#D9891
4-hydroxytamoxifen (4OHT)	Sigma Aldrich	Cat#H7904
BMP4	Peprotech	Cat#120-05ET
A83-01	Sigma-Aldrich	Cat#SML0788
Y-27632 (ROCK inhibitor)	Tocris	Cat#1254
Retinoic acid	Sigma Aldrich	Cat#R2625
2-mercaptoethanol	Sigma Aldrich	Cat#M6250
L-glutamine	Thermo Fisher	Cat#25030024
B-27 Supplement (50X)	Gibco	Cat#17504001
B-27 Supplement (50X), minus insulin	Gibco	Cat#A1895601
N-2 Supplement (100X)	Gibco	Cat#17502048
Neurobasal Medium	Gibco	Cat#21103049
DMEM/F12 Medium	Gibco	Cat#12634010
RPMI 1640 Medium, GlutaMAX Supplement	Gibco	Cat#61870036
MEM non-essential amino acids (100X)	Gibco	Cat#11140050
Sodium pyruvate (100mM)	Gibco	Cat#11360070
Accutase	Sigma Aldrich	Cat#A6964
16% Formaldehyde	Thermo Fisher	Cat#PI28906
Donkey Serum	Sigma Aldrich	Cat#D9663
EmbryoMax KSOM Medium (1X)	Sigma Aldrich	Cat#MR-106
Polyvinylpyrrolidine (PVP)	Sigma Aldrich	Cat#P0930
Triton X-100	Sigma Aldrich	Cat#T8787
Anti-mouse rabbit serum	Sigma Aldrich	Cat#M5774
Guinea pig complement	Sigma-Aldrich	Cat#234395
Acidic Tyrode’s solution	Sigma Aldrich	Cat#T1788
Bovine Serum Albumin	Gibco	Cat#15260037
Tween20	Sigma-Aldrich	Cat#P1379
Anti-Adherence Rinsing Solution	STEMCELL Technologies	Cat#07010
M2 medium	Sigma Aldrich	Cat#M7167
PMSG	Sigma Aldrich	Cat#G4527
hCG (Chorulon)	Intervet	N/A

**Critical commercial assays**		

RNeasy Mini Kit	Qiagen	Cat#74106
RNeasy Micro Kit	Qiagen	Cat#74004
RNase-Free DNase Set	Qiagen	Cat#79256
SuperScript III Reverse Transcriptase	Thermo Fisher	Cat#18080051
Universal ProbeLibrary	Roche	N/A
NEBNext Ultra II DNA Library Prep Kit for Illumina	New England Biolabs	Cat# E7645L
Chromium Next GEM Single Cell 3' Kit v3.1	10x Genomics	Cat#1000269
Chromium Next GEM Chip G Single Cell Kit	10x Genomics	Cat#1000127
NEBNext Ultra II Directional RNA Library Prep kit	New England Biolabs	Cat#E7765
NEBNext Multiplex Oligos for Illumina	New England Biolabs	Cat#E6440
Qubit dsDNA Quantification Assay Kits	Invitrogen	Cat#Q32851

**Deposited data**		

Raw and analysed data	This study	GEO:GSE232926
External re-analysed mouse embryo scRNA-seq dataset	Nowotschin et al.^53^	GEO:GSE123046
External re-analysed mouse reprogramming scRNA-seq dataset	Zhao et al.^77^	GEO:GSE114952

**Experimental models: Cell lines**		

SOX2-GFP/ GATA6-mCherry (SGGC) ESCs	Redó-Riveiro et al.^48^	N/A
OCT4-mCherry ESCs	Hashmi et al.^58^	N/A
OCT4-mCherry-H2B-Venus	This study	N/A
Esrrb KO with tetON-Esrrb transgene (EKOiE) ESCs	Festuccia et al.^82^	N/A
E14JU ESCs (E14Tg2a 129/Ola subclone)	Hamilton and Brickman^117^	N/A
DR4 mouse embryonic fibroblasts (MEFs)	Tucker et al.^118^	N/A

**Experimental models: Organisms/strains**		

C57BL/6N	Taconic	RRID:MGI:2159965
Rosa26^mT/mG^	Muzumdar et al.^45^	RRID:IMSR_JAX:007576
Pdgfra-CreER^T2^	Chung et al.^44^	RRID:IMSR_JAX:032770

**Oligonucleotides**		

See Table S6 for primer sequences used in this study	N/A	N/A

**Software and algorithms**		

BD FACSDiva	BD Biosciences	RRID:SCR_001456
FCS Express v7	De Novo Software	RRID:SCR_016431
Prism v10.1.1	GraphPad	RRID:SCR_002798
R v4.3.1	R Core Team	RRID:SCR_001905
Cell Ranger v6.1.12	10x Genomics	RRID:SCR_017344
scVelo v0.2.5 and v0.17.1	Bergen et al.^54^	N/A
scvi-tools	Gayoso et al.^74^	N/A
scanpy v1.8.2 and 1.9.1	Wolf et al.^119^	RRID:SCR_018139
Seurat v4.3.0	Hao et al.^120^	RRID:SCR_007322
BEDtools	Quinlan and Hall^121^	RRID:SCR_006646
SEACR	Meers et al.^122^	N/A
DiffBind	Ross-Innes et al.^123^	RRID:SCR_012918
deeptools	Ramírez et al.^124^	RRID:SCR_016366
SAMtools	Danecek et al.^125^	RRID:SCR_002105
IGV v2.16.2	Robinson et al.^126^	RRID:SCR_011793
pyGenomeTracks	Lopez-Delisle et al.^127^	N/A
HOMER	Heinz et al.^128^	RRID:SCR_010881
DESeq2 v1.40.2	Love et al.^129^	RRID:SCR_015687
FIJI (ImageJ) v2.14.0	Schindelin et al.^130^	RRID: SCR_002285
CellProfiler v4.2.5	Stirling et al.^131^	RRID:SCR_007358
Imaris v9.9.1	Oxford Instruments	RRID:SCR_007370
Analysed datasets and code	This study	GEO: GSE232926 Zenodo: https://doi.org/10.5281/zenodo.11231785 Github: https://github.com/brickmanlab/linneberg-agerholm-et-al-2024

**Other**		

Confocal TCS SP8 Microscope	Leica	N/A
Confocal STELLARIS Microscope	Leica	N/A
Widefield AF6000 Microscope	Leica	N/A
BD LSRFortessa 5 Cell Analyser	BD Biosciences	N/A
BD FACSAria III Cell Sorter	BD Biosciences	N/A
BD FACSymphony S6 Cell Sorter	BD Biosciences	N/A
SH800S Cell Sorter	Sony	N/A
LightCycler 480 Instrument II	Roche	N/A
A400 TapeStation	Agilent	N/A
5300 Fragment Analyzer	Agilent	N/A
Chromium Controller	10x genomics	N/A
Nunc MicroWell MiniTray	Thermo Fisher	N/A
AggreWell 400 Microwell Plates	STEMCELL Technologies	Cat#34415
NextSeq 500	Illumina	N/A
NextSeq 2000	Illumina	N/A
Qubit Fluorometer	Invitrogen	N/A

### Resource availability

#### Lead contact

Further information and requests for resources and reagents should be directed to and will be fulfilled by the lead contact, Joshua M. Brickman (joshua.brickman@sund.ku.dk).

#### Materials availability

This study did not generate new unique reagents.

#### Data and code availability

• scRNA-seq, bulk RNA-seq and CUT&Tag data has been deposited to GEO and are publicly available as of the date of publication with the accession number listed in the key resources table. This paper reanalyses existing, publicly available data and the accession numbers for these datasets are listed in the key resources table.
• The code used to generate datasets for analysis has been deposited to Zenodo and the Brickman Lab GitHub page with the links listed in the key resources table.
• Any additional information required to reanalyse the data reported in this paper is available from the lead contact upon request.

### Experimental model and study participant details

#### Mouse maintenance and embryo collection

C57BL/6N (Taconic), Rosa^mT/mG^ ^45^ and Pdgfra-CreER^T2^ ^44^ mice were housed under 12h light/dark cycles at room temperature (RT) of 22°C (± 2°C) and a humidity of 55% (±10%), with the air in the room changed 8-10 times per hour. Females were super-ovulated by intraperitoneal injection (IP) of 5 IU PMSG (Sigma Aldrich), followed by 5 IU hCG (Chorulon, Intervet) 47h later. Natural mating was set up in the evening immediately after hCG treatment and mice were checked for copulation plugs the following morning, which was determined as embryonic day (E) 0.5. Males used for mating were 8-60 weeks old, while females were 8-16 weeks old. 8-cell embryos were collected by flushing the oviducts at E2.5 using M2 medium (Sigma Aldrich). Zona pellucidae were removed from all embryos not used for chimera generation by brief incubation with acidic Tyrode’s solution (Sigma Aldrich) at RT. Concentrations used for signaling pathway modulation in embryo culture: 500 ng/mL FGF4, 1 μg/mL heparin, 1 μM PD0325901, and 1 μM JAKi. For FGF4 and heparin-treated embryos used to assess postimplantation developmental potential, these were first cultured from the 8-cell stage for 24 h in the indicated conditions followed by transfer to pseudopregnant females. Mice were healthy and not subjected to any other procedures prior to use for mating and were routinely genotyped. All work was carried out in accordance with European legislation, authorized by the Danish National Animal Experiments Inspectorate (Dyreforsøgstilsynet, license no. 2023-15-0201-01513 and 2023-15-0202-00199) and performed according to national guidelines.

#### Mouse ESC lines and culture

ESCs were maintained in naïve conditions on gelatinised tissue culture plates in N2B27 medium: 1:1 Neurobasal (Gibco) and DMEM/F-12 (Gibco), 1X B-27 (Gibco), 1X N2 (Gibco), 2mM L-glutamine (Gibco) and 100μM 2-mercaptoethanol (Gibco) supplemented with 2iLIF: 10ng/mL LIF (made in house), 3μM CHIR99021 (Axon Medchem) and 1μM PD0325901 (Sigma-Aldrich).^31^ Naïve ESCs were passaged every 2-3 days with Accutase (STEMCELL Technologies). ESCs cultured in S/L medium were maintained on gelatinised tissue culture places in GMEM basal medium (Sigma Aldrich) supplemented with 10% foetal bovine serum (FBS, Gibco), 1x MEM non-essential amino acids (Gibco), 2mM L-glutamine, 1mM sodium pyruvate, 10ng/mL LIF and 100μM 2-mercaptoethanol. ESCs were routinely tested for mycoplasma contamination and authenticated by RNA-seq and karyotyping. Concentrations used for signaling pathway modulation in nEnd culture: 1 μM JAKi, 100 μM etomoxir, and 1 mM 2DG. All cell lines used in this study were maintained under normoxic conditions at 37°C and are detailed in the key resources table.

### Method details

#### Blastocyst immunosurgery

Immunosurgery was performed on E3.5 blastocysts following removal of the zona pellucida. The embryos were placed in KSOM supplemented with 20% anti-mouse rabbit serum (Sigma Aldrich) for 1h, after which they were transferred to KSOM supplemented with 20% guinea pig complement (Sigma Aldrich).^38^ The lysed TE cells were then removed mechanically from the ICM by mouth pipetting. ICMs that disintegrated within 1-2 h following immunosurgery were not included in final analysis. ICMs isolated by immunosurgery used for transfer to pseudopregnant females were first allowed to recover for 24 h in KSOM *in vitro*.

#### Immunostaining of E4.5 embryos

Embryos between E2.5-4.5 were fixed with 4% paraformaldehyde (PFA) for 10-15min at RT with gentle rocking and washed 3 times in PBS containing 3mg/mL polyvinylpyrrolidone (PBS/PVP). The embryos were then transferred to a Nunc MicroWell MiniTray (Thermo Scientific), permeabilized (PBS/PVP and 0.25% Triton X-100) for 30min at RT and blocked with blocking buffer (PBS/PVP, 3% donkey serum, 0.1% BSA and 0.1% Triton X-100) for at least 1hr at RT or overnight at 4°C. Primary antibodies were diluted in blocking buffer and incubated overnight at 4°C in the dark, followed by 3 washes with blocking buffer for 10min each. Secondary antibodies were diluted in blocking buffer at a concentration of 1:200 and incubated for 45min at RT, followed by 3 washes with blocking buffer for 10min each. Where applicable, DAPI was added at 1:5000.

#### Immunostaining of E6.5 embryos

E6.5 embryos were timely dissected from the uterus in PBS at RT, fixed with 4% PFA for 30min at RT with gentle rocking and 3 washes in PBS for 15min. Afterwards, embryos were permeabilised in permeabilization buffer (PBS/PVP and 0.5% Triton X-100) for 30min and blocked with blocking buffer (PBS/PVP with 2% donkey serum, 0.1% BSA and 0.1% Tween20) overnight at 4°C in the dark. Primary antibodies were diluted in blocking buffer and incubated for 48h at 4°C in the dark, then washed 3 times for 15min and overnight at 4°C in the dark. Secondary antibodies were diluted in blocking buffer and incubated for 24 h at 4°C in the dark, then washed 3 times for 15min. DAPI was added to the blocking buffer at 1:5000 and incubated for 3h at RT in the dark, then washed 3 times for 15min before mounting in PBS for imaging.

#### Differentiation of ESCs to PrE

For PrE differentiation, 25x10^3^ cells per/cm^2^ cells (optimised across cell lines) were seeded on gelatinised tissue culture plates for 24 h in PrE basal medium: RPMI 1640 (Gibco) basal medium, 1X B-27 minus insulin (Gibco) and 100μM 2-mercaptoethanol. PrE basal medium was then supplemented with 10ng/mL LIF, 3μM CHIR99021 and 20ng/mL Activin A (Peprotech) (RACL medium) for an additional 4-6 days.^36^ All cells were maintained under normoxic conditions at 37°C.

#### Expansion of nEnd

At day 4-6 of PrE differentiation, cells were collected at single cell suspension and stained for PDGFRA-APC, after which PDGFRA-APC^+^ cells were isolated by FACS. E14 WT PrE cells were additionally stained for PECAM-FITC to exclude residual ESCs. SGGC PrE cells were sorted based on GATA6-mCherry expression co-stained for PDGFRA-APC. 15-20x10^3^ cells per/cm^2^ of PDGFRA-APC or GATA6-mCherry positive cells were then seeded on MEF-coated tissue culture plates in RACL medium and passaged every 3-6 days.

For 3D nEnd culture, AggreWell 400 plates were prepared as per the manufacturer’s instructions with Anti-Adherence Rinsing Solution (STEMCELL Technologies). GATA6-mCherry positive or PDGFRA-APC positive nEnd isolated by FACS were then seeded such that there were ∼50-100 cells per single AggreWell for a total of 6-12x10^4^ cells in each well containing RACL medium. Each well was then pipetted gently 2-3 times to allow even distribution of cells and the plate centrifuged at 100 x *g* for 3min. RACL medium was changed every 2 days by multiple consecutive half-medium changes. All cells were maintained under normoxic conditions at 37°C.

#### XEN cell differentiation

XEN cells were generated as previously reported^61^ by plating 1x10^4^ cells per cm^2^ of naïve ESCs on gelatinised tissue culture plates in standard XEN medium: RPMI 1640 supplemented with 15% FBS, 2mM L-glutamine and 100μM 2-mercaptoethanol. The following 2 days, medium was replaced with fresh standard XEN medium supplemented with 1μM retinoic acid (Sigma Aldrich) and 10ng/mL Activin A. On day 4, the cells were dissociated to single cell suspension with Accutase and plated at a 1:1 ratio onto fresh MEF-coated plates in standard XEN medium. XEN cells were then maintained either on MEF or gelatine-coated plates in standard XEN medium under normoxic conditions at 37°C.

#### Differentiation in TSC medium

PDGFRA-APC positive nEnd was isolated by FACS and 5x10^4^ cells seeded directly on plastic tissue culture plates in TSC medium: 30% RPMI 1640 medium supplemented with 20% FBS, 1% L-glutamine, 1mM sodium pyruvate and 100μM 2-mercaptoethanol (TSC basal medium) and 70% MEF-conditioned medium consisting of TSC basal medium cultured with MEFs for 24 h, supplemented with 25ng/mL FGF4 and 1μg/mL heparin for 6 days.^33^

#### Generation of blastoids from nEnd

DP nEnd was isolated by FACS using the OCT4-mCherry reporter cell line co-stained with PDGFRA-APC. The cells were then seeded at a density of 50 cells per microwell in an AggreWell 400 plate prepared as above in blastoid medium^57^ consisting of 50% TSC basal medium, 25% N2B27 and 25% KSOM supplemented with 12.5ng/mL FGF4, 0.5μg/mL heparin, 3μM CHIR99021, 5ng/mL BMP4 and 0.5μM A83-01. 2μM ROCKi was added for the first 24 h and blastoids were harvested after 5 days in culture.

#### Flow cytometry

Cells were dissociated to single cell suspension with Accutase, incubated with the appropriate conjugated antibody (Table S6) for 25min in PBS supplemented with 5% FBS at 4°C on ice in the dark and washed several times. DAPI was added for live/dead discrimination and cells were either analysed on a LSRFortessa (BD Biosciences) or sorted on an Aria III (BD Biosciences), Symphony S6 (BD Biosciences) or SH800 (Sony). Data was analysed using FACSDiva (BD Biosciences) and FCS Express 7 (De Novo Software).

#### Generation of chimeras

OCT4-mCherry-H2B-Venus Oct4^+^, DP and Pdgfra^+^ nEnd was isolated by FACS, after which 1 or 3 cells of either population were injected into 8-cell morulae (E2.5). Injected embryos were then cultured *in vitro* until E4.5 in KSOM under normoxic conditions at 37°C or immediately transferred back to pseudopregnant females until E6.5.

#### Immunostaining of cells

Cells were washed with PBS and fixed with 4% PFA at 37°C for 10min, permeabilised with permeabilization buffer (PBS, 1% Triton X-100) for 1h at RT and blocked with blocking buffer (PBS, 3% donkey serum, 0.1% BSA and 0.2% Triton X-100) for 1h at RT or overnight at 4°C. Primary antibodies were diluted in blocking buffer and incubated overnight at 4°C, followed by 3 washes with blocking buffer for 10min each. Secondary antibodies and DAPI were diluted in blocking buffer and incubated for 1hr at RT, followed by 3 washes with blocking buffer for 10min each.

#### RNA preparation and RT-qPCR

Total RNA was extracted using either the RNeasy Mini Kit (Qiagen) or RNeasy Micro Kit (Qiagen) and cDNA was generated using SuperScript III reverse transcriptase (Invitrogen) and Random Hexamer primers (Invitrogen). RT-qPCR was performed using the LightCycler 480 Instrument II (Roche) with the primers and probe pairs listed in Table S6 using the Universal Probe Library system. Relative concentrations were determined and normalised to the geometric mean of two housekeeping genes.

#### Library preparation for scRNA-seq and transcriptome sequencing

OCT4-mCherry 2iLIF ESCs, nEnd, 3D nEnd and nEnd to TSC were dissociated to single-cell suspension with Accutase (STEMCELL Technologies) and re-suspended in PBS supplemented with 5% FBS and 1:5000 DAPI for live/dead discrimination. DAPI negative cells were isolated by FACS for a total of 20x10^4^ cells per sample. Libraries were prepared and sequenced using 10X Genomics 3’ CellPlex Multiplexing solution. Samples were demultiplexed to GEX1 and GEX2 using Cell Ranger (v6.1.12) mkfastq. OCT4^LOF^ nEnd were isolated as described above, where libraries were prepared and sequenced using 10X Genomics Chromium Single Cell 3’ Gene Expression kit. For each sample, a cellranger multi was executed to retrieve the count matrices with mm10-2020-A as a reference genome. Configuration files are included in the GitHub link. Spliced, unspliced and ambiguous reads were determined from aligned BAM files from Cell Ranger using STARSolo v2.7.9a for each sample.

#### scRNA-seq pre-processing, filtering and quality control

The OCT4-mCherry raw dataset comprising 21,631 cells and 32,285 genes was processed by scanpy,^119^ converted to a Seurat^120^ (v4.3.0) object and filtered for cells with high mitochondrial content, low gene number and outliers, after which the dataset contained 14,788 cells. The raw UMI counts were normalized using ‘NormalizeData’ followed by ‘FindVariableFeatures’, which identified 2,000 highly variable genes. The counts were further scaled using ‘ScaleData’ with default settings to perform PCA dimensionality reduction (‘Run PCA’). Shared nearest neighbour graph was computed using the first 20 principal components, followed by identifying 14 clusters with resolution 0.6 using Louvain clustering, visualised using UMAP.

The OCT4^LOF^ raw dataset comprising 14,179 cells and 32,285 genes was similarly converted to a Seurat object and underwent filtering, after which the dataset contained 11,376 cells. The raw UMI counts were normalized using ‘NormalizeData’ followed by ‘FindVariableFeatures’, which identified 2,000 highly variable genes. The counts were further scaled using ‘ScaleData’ with default settings to perform PCA dimensionality reduction (‘Run PCA’). Shared nearest neighbour graph was computed using the first 20 principal components, followed by identifying 8 clusters with resolution 0.2 using Louvain clustering, visualised using UMAP.

#### RNA velocity

STARSolo output matrices were merged with the processes count matrix based on common cell barcodes. RNA velocity analysis was performed using scVelo^54^ following the recommended workflow. n_neighbors and n_pcs to 20 were adjusted and 2,000 highly variable genes used with dynamical modelling to estimate cellular dynamics. The velocities were projected on already computed UMAP visualisation. To infer latent time that matched the experimental setup, we set cluster 7 (DP) as a starting point.

#### Integration of scRNA-seq with published data

Based on an extensive benchmarking review on integration tools,^132^ we integrated the Nowotschin et al.^53^ and Zhao et al.^77^ datasets using scVI.^74^ We subset Nowotschin et al.^53^ to E3.5 and E4.5 stages and merged them with our experiment. Integration with Nowotschin et al.^53^ was done based on a subset of 2,000 highly variable genes, whereas 3,000 highly variable genes were used for the Zhao et al.^77^ dataset. Raw counts were used with the following settings: dropout_rate = 0.2, dispersion = gene and gene_likehood = nb to train scVI. The integration was validated by performing trajectory analysis using PAGA^78^ on the learned latent space. Further details are deposited on GitHub.

#### CUT&Tag

CUT&Tag was performed as previously described^83^^,^^133^ with slight alterations. All samples were isolated by FACS based on OCT4-mCherry and PDGFRA-APC expression where 25x10^5^ cells per sample replicate was used for downstream processing. Primary antibody incubation was done at 4°C overnight, Tn5 was purchased from EMBL Heidelberg and DNA precipitation was performed over the weekend at -80°C. DNA was amplified with 14 PCR cycles and the samples were sequenced paired-end on an Illumina NextSeq 500. Downstream analysis was performed using BEDtools^121^ to determine BedGraph coverage, peaks were then called against an IgG control using SEACR^122^^,^^134^ using the ‘stringent’ parameter, and consensus peaks were determined using DiffBind,^123^ visualized with deeptools.^124^ BAM files were merged for visualisation using SAMtools,^125^ IGV^126^ and pyGenomeTracks^127^ and annotation of peaks was performed using HOMER.^128^

#### Bulk RNA-seq

RNA was purified using the RNeasy Mini Kit (Qiagen) with a 15min treatment of RNase-Free DNase (Qiagen) at RT. Quality of the RNA was determined using a TapeStation (Agilent). 1μg of RNA per sample was used for library preparation with the NEBNext Ultra II Directional RNA Library Prep Kit (New England Biolabs) and the samples were sequenced single-end on an Illumina NextSeq 2000. Differential expression analysis was performed using DESeq2.^129^

### Quantification and statistical analysis

#### Quantification and imaging analysis

Cells were imaged using either a confocal Leica TCS SP8, confocal Leica STELLARIS or widefield Leica AF6000 microscope. Image analysis was carried out using FIJI (ImageJ),^130^ Imaris v9.9.1 (Oxford Instruments) and CellProfiler v4.2.5.^131^ All image quantification was performed on randomised single optical sections of original images from multiple images taken across different biological replicates. Maximum projections were used for visualisation purposes only.

#### Statistical analysis and reproducibility

All embryo treatments used for statistical analysis were performed using a minimum of two independent litters at different times, where control embryos were littermates to the corresponding treatment for a given experiment. Statistical tests were performed using R and GraphPad Prism 10. Unless otherwise stated, experiments were performed with three biological replicates (*n* = 3) and significance was defined as: not significant (ns) ≥ 0.05; ^∗^*P* < 0.05; ^∗∗^*P* < 0.01; ^∗∗∗^*P* < 0.001.

## Supplemental information

Table S1. Overview of *n* values, lineage allocation and success rates for embryo experiments, related to Figures 1 and S1

Table S2. Analysis of scRNA-seq dataset for nEnd to TSC differentiation, related to Figures 3 and S5

Table S3. Analysis of scRNA-seq dataset for nEnd, 3D nEnd, and 2iLIF ESCs, related to Figures 5, S6, and S7

Table S4. Analysis of CUT&Tag for nEnd subpopulations, related to Figures 7 and S8

Table S5. Normalized count table for bulk RNA-seq of nEnd subpopulations, related to Figures 7 and S8

Table S6. Details of antibodies and primers used, related to STAR Methods
